# Inherited mitochondrial optic neuropathies

**DOI:** 10.1136/jmg.2007.054270

**Published:** 2008-11-17

**Authors:** P Yu-Wai-Man, P G Griffiths, G Hudson, P F Chinnery

**Affiliations:** 1 Mitochondrial Research Group, The Medical School, Newcastle University, Newcastle upon Tyne, UK; 2 Department of Ophthalmology, Royal Victoria Infirmary, Newcastle upon Tyne, UK; 3 Institute of Human Genetics, Newcastle University, Newcastle upon Tyne, NE1 3BZ, UK

## Abstract

Leber hereditary optic neuropathy (LHON) and autosomal dominant optic atrophy (DOA) are the two most common inherited optic neuropathies and they result in significant visual morbidity among young adults. Both disorders are the result of mitochondrial dysfunction: LHON from primary mitochondrial DNA (mtDNA) mutations affecting the respiratory chain complexes; and the majority of DOA families have mutations in the *OPA1* gene, which codes for an inner mitochondrial membrane protein critical for mtDNA maintenance and oxidative phosphorylation. Additional genetic and environmental factors modulate the penetrance of LHON, and the same is likely to be the case for DOA which has a markedly variable clinical phenotype. The selective vulnerability of retinal ganglion cells (RGCs) is a key pathological feature and understanding the fundamental mechanisms that underlie RGC loss in these disorders is a prerequisite for the development of effective therapeutic strategies which are currently limited.

Mitochondrial disorders are a major cause of chronic human disease with an estimated prevalence of 1 in 10 000 in the UK and a further 1 in 200 individuals being at-risk mutational carriers.^1^ ^2^ Ocular involvement is a prominent feature in this group and often points towards the underlying mitochondrial aetiology, which allows for a more targeted diagnostic approach. Optic nerve dysfunction can be the presenting and only ophthalmological manifestation causing the two most common inherited optic neuropathies encountered in clinical practice, Leber hereditary optic neuropathy (LHON) and autosomal dominant optic atrophy (DOA), which are the focus of this review. In the majority of cases, the pathology in LHON and DOA is limited to a highly specialised group of cells within the eye, the retinal ganglion cells (RGCs), but the phenotype associated with these two conditions is expanding, providing important insights into possible disease pathways leading to optic nerve degeneration and visual failure.

## LEBER HEREDITARY OPTIC NEUROPATHY

### LHON mutations

LHON (OMIM 535000) was first described as a distinctive clinical entity in 1871 by the German ophthalmologist Theodore Leber (1840–1917).^3^ He reported a characteristic pattern of visual loss among members of four families and his observations were subsequently confirmed in pedigrees from different populations.^4^^–^6 These early studies highlighted several of the salient features of LHON including the maternal transmission of the disease, the predilection of males to lose vision, and the almost exclusive involvement of the optic nerve. The non-Mendelian pattern of inheritance was only fully explained in 1988 when LHON became the first human disease proven to be caused by a point mutation (m.11778G>A) within the mitochondrial genome.^7^ Over 95% of LHON pedigrees are now known to harbour one of three mitochondrial DNA (mtDNA) point mutations: m.3460G>A, m.11778G>A and m.14484T>C, which all involve genes encoding complex I subunits of the mitochondrial respiratory chain.^8^ In a meta-analysis of 159 pedigrees from Northern Europe and Australia, m.11778G>A was the most prevalent mutation but there is considerable variation in the relative frequency of these three primary LHON mutations worldwide (table 1). The predominance of m.11778G>A is even more pronounced in the Far East where it accounts for ∼90% of all cases,^9^ ^10^ and although m.14484T>C is relatively rare, it is the most common mutation found among French Canadians (87%) as a result of a founder event.^11^ ^12^ Primary mutations have not been identified in a small minority of clinically diagnosed LHON patients, the most likely explanation being that rare pathogenic mtDNA variants are segregating in these families.^13^ Disease causing mutations have been identified in a proportion of these cases, while other putative LHON mutations require further confirmation as they have only been found in singletons or a single family (table 1).

**Table 1:** Pathogenic mtDNA mutations associated with Leber hereditary optic neuropathy

	Mutation	Gene	Prevalence (%)	Reference
Primary			>95	
	m.3460G>A	*MT-ND1*	13	220, 221
	m.11778G>A	*MT-ND4*	69	7
	m.14484T>C	*MT-ND6*	14	32, 222
Rare			<5	
	m.3376G>A	*MT-ND1*		45
	m.3697G>A			46
	m.3733G>A			223
	m.4160T>C			41
	m.4171C>A			224
	m.11696G>A	*MT-ND4*		40
	m.11253T>C			225
	m.10663T>C	*MT-ND4L*		226
	m.12848C>T	*MT-ND5*		227
	m.13730G>A			228
	m.14568C>T	*MT-ND6*		229
	m.14279G>A			230
	m.14459G>A			42–44
	m.14482C>G			231
	m.14495A>G			232
	m.14498C>T			233
	m.14568C>T			234
	m.14596A>T			40

### Epidemiology

LHON is the most common of the primary mtDNA diseases, with a minimum prevalence of 1 in 31 000 affected individuals in the North East of England and 1 in 8500 carriers being at-risk of visual loss.^14^ Fairly similar figures have been reported in other Caucasian populations, with an LHON prevalence of 1 in 39 000 in the Netherlands and 1 in 50 000 in Finland.^15^ ^16^ About 2% of visually impaired people on the blind register in Australia are also reported to suffer from LHON.^17^ The peak age of onset in LHON is between the age of 15–30 years and 95% of carriers who will experience visual failure will do so before the age of 50 years (table 2). However, visual deterioration can occur anytime during the first to the seventh decade of life and LHON should be part of the differential diagnosis for all cases of bilateral, simultaneous or sequential optic neuropathy, irrespective of age and especially in male patients.^18^ ^19^ Except for one report which found a slight increase in the age of onset in females carrying the m.11778G>A mutation,^20^ it is generally accepted that neither gender nor mutational status significantly influences the timing and severity of the initial visual loss.^11^ ^21^^–^23 Affected individuals are often aware of other affected family members, but up to 40% have no family history. These most likely represent cases where family history is difficult to trace back, given that de novo mutations are rare in LHON.^14^ ^24^

**Table 2:** Lifetime risk of visual failure for Leber hereditary optic neuropathy carriers and recovery rates

	Pedigrees (n)	Median onset	Male: female ratio	Visual recovery (%)	Reference
m.3460G>A	9	29 years	2.3:1	22	22
	8	20 years	4.3:1	25	20
m.11778G>A	49	28 years	4.5:1	4	21
	66	24 years	3.7:1	25	20
	10	29 years	5.3:1	25	28
m.14484T>C	17	27 years	2.1:1	37	23
	23	19 years	7.7:1	58	11

### Clinical features

#### Pre-symptomatic phase

Fundal abnormalities such as telangiectatic vessels around the optic discs and variable degrees of retinal nerve fibre layer oedema have been documented in some asymptomatic carriers, and these can fluctuate with time. Using optical coherence tomography imaging, thickening of the temporal retinal nerve fibre layer was found in a proportion of unaffected LHON carriers, which provides further evidence that the papillomacular bundle is particularly vulnerable in this disorder.^25^ ^26^ On more detailed psychophysical testing, some individuals also exhibited subtle impairment of optic nerve function including loss of colour vision affecting mostly the red–green system, reduced contrast sensitivity, and subnormal visual electrophysiological parameters.^27^

### Acute phase

**Figure 1:**
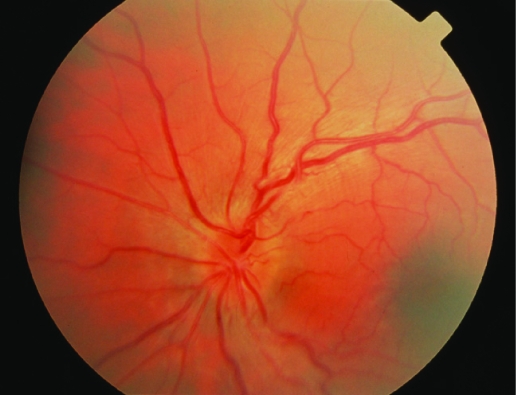
Acute fundal appearance in Leber hereditary optic neuropathy showing disc hyperaemia, swelling of the parapapillary retinal nerve fibre layer and retinal vascular tortuosity.

LHON carriers remain asymptomatic until they experience blurring or clouding of vision in one eye. In the vast majority of cases, visual dysfunction is bilateral, the fellow eye becoming affected either simultaneously (25%) or sequentially (75%), with a median inter-eye delay of 6–8 weeks.^20^ Rare cases of unilateral optic neuropathy in LHON have been reported, with the fellow eye remaining unaffected over a follow-up period of up to 16 years.^28^ ^29^ Visual acuity reaches a nadir 4–6 weeks after disease onset and it is severely reduced to 6/60 or less. The characteristic field defect is a steep-sided central or centrocaecal scotoma and this can be formally documented using Goldmann or kinetic perimetry. Other clinical features include the early impairment of colour perception but, importantly, pupillary reflexes are preserved and patients usually report no pain on eye movement. Ocular examination during the acute stage provides other diagnostic clues and in classical cases the following abnormalities can be observed: vascular tortuosity of the central retinal vessels, swelling of the retinal nerve fibre layer, and a circumpapillary telangiectatic microangiopathy (fig 1). However, it must be stressed that in ∼20% of LHON cases, the optic disc looks entirely normal in the acute phase.^30^ ^31^

### Chronic phase

The retinal nerve fibre layer gradually degenerates and after 6 months, optic atrophy is a universal feature. If a patient is only seen at this stage, it can be difficult to exclude other compressive, infiltrative and inflammatory causes of a bilateral optic neuropathy, especially if there is no clear maternal family history. In these cases, neuroimaging of the anterior visual pathways is mandatory while awaiting the results of molecular genetic testing.

### Visual recovery

Visual recovery is observed in some patients even several years following disease onset. but the chances of improvement are influenced by the patient’s mutational status, being least with the m.11778G>A mutation, highest with the m.14484T>C mutation, and the m.3460G>A mutation having an intermediate visual prognosis (table 2). The recovery in visual parameters is not only restricted to visual acuity, but can also include the development of small islands of normal field (fenestrations) within the central scotoma or a reversal of dyschromatopsia.^28^ ^32^ ^33^ Positive prognostic factors for visual improvement are an early age of onset (<20 years), subacute presentation with slow progression of the visual deficits, and large optic nerve head surface area.^28^ ^34^ However, LHON is a devastating disorder with the majority of patients showing no functional improvement and remaining within the legal requirement for blind registration.

### Associated features

Although visual failure is the defining feature in this mitochondrial disorder, cardiac arrhythmias and neurological abnormalities such as postural tremor, peripheral neuropathy, non-specific myopathy and movement disorders have been reported to be more common in LHON compared to controls.^35^^–^39 These are rarely clinically significant but a small number of LHON pedigrees do have severe neurological deficits (spastic dystonia, ataxia and juvenile onset encephalopathy) in addition to the optic neuropathy. These “LHON plus” syndromes have been linked to various mtDNA mutations in isolated pedigrees from Holland, Australia and North America: A11696G and/or T14596A,^40^ T4160C,^41^ and G14459A,^42^^–^44 respectively. Two mtDNA complex I mutations point mutations, m.3376G>A^45^ and m.3697G>A,^46^ have also recently been identified in individuals with overlap clinical features of both LHON and MELAS (mitochondrial encephalomyopathy, lactic acidosis, and stroke-like episodes). Interestingly, a significant minority of Caucasian LHON carriers, predominantly females with the m.11778G>A mutation, develop clinical and neuroimaging features indistinguishable from multiple sclerosis (MS), including unmatched oligoclonal bands in the cerebrospinal fluid (Harding’s disease).^47^^–^50 It is currently not known whether the prevalence of this MS-like illness in LHON is higher than expected due to the chance occurrence of these two disorders, and although controversial, some investigators have argued for a potential role of autoimmunity in the pathophysiology of this mitochondrial disorder.^51^^–^55

### Diagnosis

A tentative diagnosis of LHON can usually be made on clinical grounds, especially if classical ophthalmological features are present and a clear maternal history is elicited. Molecular genetic testing on a blood DNA sample, however, remains the gold standard and will confirm that the patient harbours one of the three primary mtDNA LHON mutations, with implications for future genetic counselling. If indicated, electrophysiological studies, including pattern electroretinograms (PERGs) and visual evoked potentials (VEPs), can be carried out to exclude retinal pathology and confirm optic nerve dysfunction.^56^ An electrocardiogram is also recommended to exclude a pre-excitation syndrome which has been documented in LHON, although such a finding is rare and does not require any intervention in the absence of cardiac symptoms.^30^ ^31^ Computed tomography (CT) and magnetic resonance imaging (MRI) scans are usually normal in LHON, but there are reports of non-enhancing high signals within the optic nerve and sheath distension, secondary to slight oedema or gliosis in the atrophic phase.^57^^–^62

### Biochemical features

Oxidative phosphorylation (OXPHOS) provides for most of the cell’s adenosine triphosphate (ATP) requirements and this is achieved by a chain of five respiratory complexes situated on the inner mitochondrial membrane. Since all three primary LHON mutations involve complex I subunits, one would expect respiratory chain function to be compromised, leading to a deficit in ATP synthesis and RGC degeneration as a consequence of energy failure. However, both in vitro and in vivo biochemical studies have produced conflicting results regarding the extent of respiratory chain dysfunction in LHON (table 3). In a small number of in vivo studies using phosphorus magnetic resonance spectroscopy (31P-MRS), the most consistent defect of mitochondrial function was identified in persons with the m.11778G>A mutation and none among those with the m.3460G>A mutation.^63^^–^67 A striking conclusion from all these biochemical studies is that no significant difference between affected and unaffected individuals with a disease causing LHON mutation could be demonstrated. But as none of these studies have been performed directly on RGCs and the causative biochemical mechanisms could be highly tissue-specific, further studies are warranted.

**Table 3:** Respiratory chain dysfunction in Leber hereditary optic neuropathy

MtDNA mutation	In vitro^88^,^235^^–^249			In vivo^63^^–^67
	Complex I activity (%)	Respiratory rate (%)	ATP synthesis (%)	31P MRS (%)
m.3460G>A	60–80	30–35	90	0–40
m.11778G>A	0–50	30–50	35	75
m.14484T>C	0–65	10–20	90	50

ATP, adenosine triphosphate; 31P-MRS, phosphorus magnetic resonance spectroscopy.

% decrease relative to controls.

### Neuropathology

These functional studies also raise important issues regarding the cell specific ocular pathology in LHON which is limited to the RGC layer, with sparing of the retinal pigment epithelium and photoreceptors. There is pronounced cell body and axonal degeneration, with associated demyelination and atrophy observed from the optic nerves to the lateral geniculate bodies. Experimental data indicate impaired glutamate transport,^68^ oxidative stress^69^ ^70^ and increased mitochondrial reactive oxygen species (ROS)^71^ within RGCs and support an apoptotic mechanism of cell death.^72^ ^73^ LHON patients also have reduced α-tocopherol/lipid ratios and high levels of 8-hydroxy-2-deoxygaunosine in blood leucocytes, both biological markers of increased free radical production.^74^ ^75^ However, the selective vulnerability of RGCs in LHON still remains unexplained, and this area of research has been greatly hampered by the lack of access to diseased human tissues, the retina and optic nerve not being amenable to biopsies.

### Animal models

The development of faithful animal models in LHON is therefore critical but there is still no murine model where the primary LHON mutations have been successfully introduced within the mitochondrial genome. In spite of these technical challenges, significant advances have been made over the past decade and there are currently three experimental paradigms, all of which disrupt OXPHOS and recapitulate the optic nerve degeneration seen in LHON: (1) intravitreal injection of a respiratory chain poison such as rotenone^76^; (2) downregulation of nuclear encoded complex I subunits (for example, NFUFA1) with specific mRNA-degrading ribozymes^77^; and (3) allotropic expression of mutant subunits (for example, MTND4) which are then imported into the mitochondria.^78^

### Incomplete penetrance

**Figure 2:**
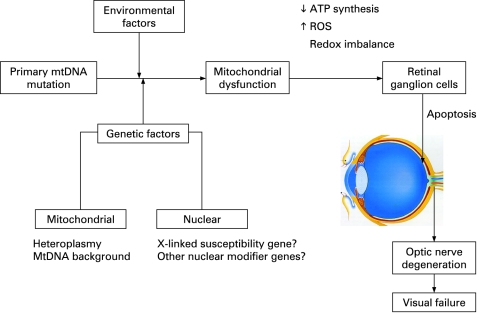
Secondary factors interacting with the primary mtDNA Leber hereditary optic neuropathy mutation to precipitate visual loss. ATP, adenosine triphosphate; ROS, reactive oxygen species.

An intriguing feature of LHON is that only ∼50% of males and ∼10% of females who harbour one of the three primary mutations actually develop the optic neuropathy. This incomplete penetrance and predilection for males to lose vision imply that additional genetic and/or environmental factors must modulate the phenotypic expression of LHON (fig 2). Alternatively, the gender bias could also result from a combination of subtle anatomical, hormonal and physiological variations between males and females.

### Mitochondrial genetic factors

#### Heteroplasmy

Depending on their metabolic demands, cells can contain anywhere between 100–10 000 mitochondria, and with 2–10 mtDNA molecules in each mitochondrion, this results in a very high copy number per cell. In most LHON pedigrees, the primary mutation is homoplasmic—that is, every mtDNA molecule harbours the mutant allele. By contrast, 10–15% of LHON carriers are thought to be heteroplasmic, with one mtDNA sub-population carrying the wild type allele.^14^ ^20^ ^79^ Although limited and retrospective, the available data suggest that heteroplasmy contributes to incomplete penetrance, with the risk of blindness being minimal if the mutational load is <60%.^80^ However, quantifying the level of heteroplasmy for the purpose of pre-symptomatic testing is limited as the majority of individuals with a LHON mutation are homoplasmic.

#### MtDNA haplogroups

MtDNA accumulates mutations ∼10 times faster than nuclear genome, resulting in a high degree of polymorphism.^81^ Because human mtDNA is strictly maternally inherited and does not recombine, polymorphisms have accumulated sequentially along radiating female lineages as women migrated out of Africa into the different continents ∼150 000 years ago.^82^ Reflecting its evolution, a number of stable polymorphic variants cluster together in specific combinations referred to as haplogroups, with individuals of European ancestry belonging to one of nine haplogroups: H, I, J, K, T, U, V, W and X.^83^ ^84^ A recent meta-analysis of 159 European LHON pedigrees indicated that the risk of visual loss for the three primary LHON mutations is influenced by the mtDNA background.^85^ The risk of visual failure was greater when the m.11778G>A and m.14484T>C mutations arose on haplogroup J, whereas individuals with the m.3460G>A mutation were more likely to experience visual loss if they belonged to haplogroup K. On the other hand, individuals with the m.11778G>A mutation had a lower risk of visual loss when the mutation arose on haplogroup H. Haplogroups H, J and K are all defined by non-synonymous, polymorphic substitutions in the *MT-CYB* gene which codes for cytochrome *b*, the only mitochondrially encoded subunit of complex III. Recent experimental data support the existence of stable respiratory chain supercomplexes, one of which consists of a complex I monomer physically interacting with a complex III dimer. Although speculative, the haplogroup associated amino acid substitutions within cytochrome *b* could therefore influence the risk of visual failure by modulating the biochemical consequences of the primary LHON mutations through an effect on the stability of these putative I-III supercomplexes.^85^^–^87 In support of this hypothesis, cybrid cell lines carrying the m.11778AG>A mutation on a haplogroup J background had a lower oxygen consumption and a longer doubling time compared to non-haplogroup J cell lines.^88^ However, haplogroup J was not found to further impair mitochondrial OXPHOS in the brain and skeletal muscle of patients harbouring the m.11778G>A mutation with 31P-MRS measurements,^66^ and a study of South-East Asian LHON pedigrees found no association between specific mtDNA haplogroups and the risk of visual loss.^89^ These contradictory findings reflect the need for additional studies to clarify the significance of the mtDNA background on LHON penetrance.

### Nuclear genetic factors

The predominance of affected males in LHON cannot be explained by mitochondrial inheritance and segregation analysis suggests the existence of a recessive X-linked susceptibility gene acting in synergy with the mtDNA mutation to precipitate the optic neuropathy.^90^^–^92 In the *Bu and Rotter* model, the development of blindness in males is consistent with the simultaneous inheritance of an X-linked visual loss allele and the primary LHON mutation, whereas females are affected either if they are homozygous at the susceptibility locus (40%) or heterozygous with skewed X chromosome inactivation of the wild-type allele (60%). Several studies have, however, failed to demonstrate any skewed X chromosome inactivation in affected female carriers, albeit in blood leucocytes and not in RGCs which are the affected tissues in LHON.^93^^–^95 Initial attempts to identify this X-linked susceptibility locus by standard linkage analysis were unsuccessful,^96^^–^99 but two recent studies using a larger number of more extensively defined LHON pedigrees found two overlapping disease loci with highly significant LOD scores at Xp21–Xq21^100^ and Xq25–27.2.^101^ Although the actual causative gene in this region of interest has not yet been identified, a high risk haplotype [DXS8090(166)-DXS1068(268)] at Xp21 was defined which increased the risk of visual failure ∼35-fold for the m.11778G>A and m14484T>C mutations but not for m.3460G>A.^100^ The possibility of other autosomal nuclear modifier genes in LHON has not been excluded and the genetic aetiology of LHON might prove even more complex, with epistatic interaction of these multiple nuclear susceptibility loci and genetic heterogeneity.

### Environmental factors

Five pairs of monozygotic twins harbouring a primary LHON mutation have been reported in the literature, and in two cases the twins have remained discordant.^20^ ^21^ ^24^ ^102^^–^104 Although there is always the possibility that the unaffected sibling will lose vision later on in life, the existence of discordant monozygotic twins strongly suggests that environmental factors also contribute to penetrance. There are several reports of an increased risk of visual loss among LHON carriers with high tobacco and alcohol consumption,^105^^–^108 but the largest case–control study to date has failed to confirm this association.^109^ There are also anecdotal reports of nutritional deprivation, exposure to industrial toxins, antiretroviral drugs, psychological stress or acute illness precipitating the onset of blindness in LHON.^108^ ^110^^–^112 Of note, in some pedigrees the penetrance of LHON seems to be decreasing, falling to 1% and 9% in younger generations of two large, multi-generational pedigrees from Australia^113^ and Brazil,^108^ ^114^ respectively. Both carry homoplasmic levels of the m.11778G>A mutation and this phenomenon has been ascribed to improved environmental and socio-economic factors. However, a much larger epidemiological study of 3613 LHON carriers from multi-generational pedigrees failed to detect a change in the penetrance of the three primary LHON mutations. The role of environmental triggers in LHON remains largely unanswered and more robust epidemiological data are needed, which will necessitate a multicentre collaborative effort in order to collect sufficient number of subjects for analysis.

### Treatment

No generally accepted measures have been shown to either prevent or delay the onset of blindness in LHON, but for general health reasons LHON carriers should be advised to moderate their alcohol intake and stop smoking. In two small case series, oral administration of idebenone, a synthetic analogue of coenzyme Q10, and vitamin B12 and C supplementation led to faster and greater visual recovery among affected individuals.^115^ ^116^ However, a recent study has not found any improved visual prognosis from idebenone and multivitamin supplementation, and properly conducted treatment trials are needed before such a regimen can be advocated.^117^ The use of brimonidine eye drops, which is thought to have anti-apoptotic properties, was also unsuccessful in preventing second eye involvement in recently affected patients with unilateral optic neuropathy.^118^ The long term management of visually impaired patients remains supportive, with provision of visual aids and registration with the relevant social services.

### Genetic counselling

It is important to stress to LHON carriers that it is not possible to predict accurately whether or when they will become affected. Despite these caveats, the two main predictive factors for visual failure remain age and gender, with males having about a 50% lifetime risk of blindness compared to only 10% for females, and these approximate figures can be further refined based upon the patient’s age. From published age dependent penetrance data, most patients experience visual loss in their late teens and 20s and the probability of becoming affected decreases with increasing age, being minimal once past the age of 50 years (table 2). Once a primary LHON mutation has been identified in a proband, other maternally related family members can be offered molecular genetic testing to exclude the possibility of a de novo mutation, which is rare. Since LHON shows strict maternal inheritance, male carriers can be reassured that none of their children will inherit the mtDNA mutation whereas female carriers will transmit the pathogenic mutation to all of their offspring. Since most mothers are homoplasmic, their children will only harbour the mutant species, but the situation is more complex for a heteroplasmic mother as she could transmit a higher or a lower level of the mutation to a particular offspring, which will impact on the latter’s risk of visual failure. Although the mutant level can be determined and there is evidence that a mutational threshold of ∼60% in blood is necessary for disease expression, genetic counselling for these unaffected heteroplasmic carriers remains difficult. For similar reasons, the prenatal genetic testing of heteroplasmic women with amniocentesis or chorionic villus sampling (CVS) would be difficult to interpret.

## DOMINANT OPTIC ATROPHY

### Clinical features

The clinical features of DOA (OMIM 165500) were first described in one British family by Batten in 1896^119^ ^120^; the phenotype was further clarified by Kjer in his extensive study of Dutch families in the 1950s,^119^ ^120^ distinguishing it from LHON with which the disease was often confused. The prevalence of DOA is not well established and robust estimates based on molecular confirmation are not available, although a historical figure of 1 in 50 000 among Caucasians is often quoted in the literature.^121^ It is thought to be the most common inherited optic neuropathy in the Netherlands, with a population frequency of 1 in 12 000, and this much higher prevalence has been linked to a mutational founder event.^122^

The onset of symptoms in DOA is relatively insidious. In pre-molecular case series, 13–25% of patients with optic atrophy were visually asymptomatic and were only identified through contact tracing via other affected family members.^123^ ^124^ Classically, the visual decline starts in the first two decades of life, but there is a pronounced inter- and intra-familial variability in the severity of visual symptoms, which makes genetic counselling difficult. Visual acuity can range from 6/6 to the detection of hand movement only, and the rate of progression of visual loss is not easy to predict, with 19–50% of patients experiencing further, albeit slow, deterioration on long term follow up.^125^^–^129 Although the overall visual prognosis is better when compared to LHON, with a mean visual acuity of 6/24–6/36, DOA results in significant visual impairment with about half of all affected individuals failing the driving standards and 13–46% registered as legally blind.^130^^–^132

The predominant colour defect in DOA is a generalised dyschromatopsia, involving both the blue–yellow and red–green axes, with a minority of patients having pure tritanopia (<10%), which was once considered to be a pathognomonic feature of DOA.^133^ Central, centrocaecal and paracentral scotomas are the most common field abnormalities with sparing of the periphery, findings consistent with the primary involvement of the papillomacular bundle in this condition. Interestingly, as in LHON, there is usually no afferent pupillary defect, suggesting that the retino-tectal fibres sub-serving the pupillary light reflex are less susceptible to the downstream effects of both the LHON mtDNA mutations and the causative nuclear genetic defects in DOA.^134^ However, both magnocellular and parvocellular RGC pathways seem to be similarly affected, although this requires further investigation.^127^ ^131^

**Figure 3:**
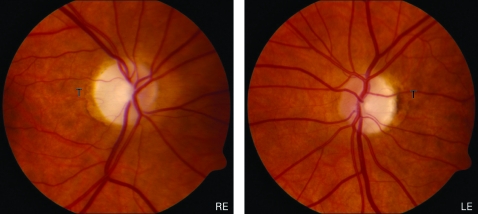
Typical fundal appearance in dominant optic atrophy showing bilateral optic disc pallor more marked in the temporal quadrant (LE, left eye; RE, right eye; T, temporal quadrant).

The optic disc pallor in DOA falls into two main categories: diffuse pallor involving the entire neuro-retinal rim in about half of all cases, and a temporal wedge in the remainder (fig 3).^123^ ^135^ However, disc pallor can be subtle and 29% of affected patients had normal looking optic discs in one case series, highlighting the need to look carefully for other features of optic nerve dysfunction when assessing patients with a possible diagnosis of DOA.^132^ Other common optic disc findings include saucerisation (79%), peripapillary atrophy (69%) and a cup to disc ratio >0.5 (48%).^131^ ^135^ ^136^ The measurement of circumpapillary retinal nerve fibre layer thickness using optical coherence tomography (OCT) could also prove a useful adjunct in the diagnostic work-up of DOA, with recent studies showing a typical profile with bilateral symmetrical thinning around the optic disc, most pronounced in the temporal quadrant.^137^ ^138^

### Ocular pathology

Postmortem studies of two patients with DOA identified similar histopathological changes, with diffuse atrophy of the RGC layer, loss of myelin and fibrillary gliosis along the anterior visual pathways extending to the lateral geniculate body.^139^ ^140^ More recent MRI data from patients with DOA have also confirmed significant tissue loss and thinning of the optic nerve along its entire length.^141^ Although less pronounced, the underlying ocular pathology in DOA is therefore remarkably similar to LHON, with the primary loss of RGCs leading to ascending optic atrophy.

Visual electrophysiological findings are well documented in DOA and provide additional evidence for the primary loss of RGCs and the sparing of the outer retinal layers.^133^ ^142^ ^143^ It can therefore be a useful ancillary test when determining affected status in borderline DOA cases and also in excluding a primary retinal process such as early cone dystrophy. VEPs are either absent or, if traces are recordable, they are of low amplitudes with abnormal latencies. PERGs can be within the normal range in up to 40% of clinically affected individuals but usually demonstrate an abnormal P50:N95 ratio, with selective depression of the N95 negative wave amplitude confirming a primary optic nerve pathology. Additional involvement of the P50 component correlates with the severity of visual loss, but PERGs are not extinguished even in cases where visual acuity is reduced to detection of hand movements or worse.

### Molecular genetics

The majority of DOA families show linkage to the *OPA1* locus at 3q28–q29, and in 2000 two independent research groups identified pathogenic mutations in the *OPA1* gene.^144^ ^145^ The proportion of *OPA1* positive families is ∼60% (range 32–89%), the lower detection rates in some of these case series reflecting the inclusion of singleton cases, a heterogeneous group that is more likely to include non-inherited forms of optic neuropathy, and the use of less sensitive mutation screening protocols such as single strand conformational polymorphism (SSCP) analysis.^146^ ^147^ Interestingly, a recent report suggested that large scale rearrangements of entire *OPA1* coding regions could account for up to 20% of all *OPA1* negative cases.^148^

The causative nuclear defects in the remaining families with DOA have not yet been identified, but a small number of families have been mapped to other chromosomal loci—OPA3, OPA4, OPA5 and OPA7, of which only the *OPA3* gene has been characterised (table 4). The *OPA3* gene was originally identified in eight Iraqi Jewish families with an autosomal recessive form of optic atrophy, associated with neurocognitive deficits, elevated urinary excretion of 3-methyl glutaconic acid, and increased plasma 3-methylglutaric acid levels (type III 3-methylglutaconic aciduria or Costeff syndrome).^149^^–^151 However, pathogenic mutations in the *OPA3* gene have also been identified in two French families segregating both DOA and premature cataract in an autosomal dominant mode of inheritance (ADOAC).^152^ ^153^ The Opa3 protein is located in the mitochondrial inner membrane but its exact function remains to be clarified. Preliminary findings in cultured fibroblasts from a patient with ADOAC indicate an increased susceptibility to apoptosis, and one can speculate that a similar mechanism is leading to RGC dysfunction via disruption of the mitochondrial respiratory chain.^152^ ^154^ ^155^

**Table 4:** Dominant optic atrophy loci reported in *OPA1* negative families

	OMIM	Reported locus		Causative gene	Families (n)	Clinical features	Reference
OPA-3	606580	19q13.2–q13.3		*OPA3*	2	Optic atrophy + premature cataract	152
OPA-4	605293	18q12.2–q12.3		Unknown	1	Optic atrophy*	250
OPA-5	610708	22q12.1–q13.1		Unknown	2	Optic atrophy*	251
OPA-7	–	16q21–q22		Unknown	1	Optic atrophy + deafness	252

*Similar clinical phenotype to *OPA1* positive families.

### *OPA1* mutations

The *OPA1* gene consists of 30 exons spanning over 100 Kb of genomic DNA and it codes for a 960 amino acid, dynamin related GTPase protein located within the inner mitochondrial membrane. Alternative splicing of exons 4, 4b and 5b result in eight different mRNA isoforms, and both their functional relevance and subcellular localisation are currently being investigated.^156^ Over 140 pathogenic mutations have been identified and these cluster in two specific regions: the GTPase region (exons 8–15) and the C-terminus which is the proposed site of the GTPase effector domain. The majority of *OPA1* mutations (∼50%) lead to premature termination codons (PTCs) as a result of nonsense mutations or frameshifts from small insertions, deletions or splice site mutations (e*OPA1* database at http://lbbma.univ-angers.fr/lbbma.php?id = 9).^157^ These truncated mRNAs are unstable and get degraded by specific pathways (nonsense mediated mRNA decay), which are in-built protective cellular mechanisms against mutant proteins with possible dominant negative effects.^158^^–^160 The reduced Opa1 protein expression levels observed in these cases support the role of haploinsufficiency in DOA and this is further substantiated by one family with a microdeletion resulting in complete loss of one copy of the *OPA1* gene.^161^ However, ∼30% of *OPA1* mutations are missense mutations within or close to the GTPase domain and these could exert their pathogenic effect via a deleterious, gain of function mechanism.^162^^–^164

### Gene expression

The spatial localisation and expression pattern of the Opa1 protein have been examined in a wide range of post-mitotic human and murine tissues. The Opa1 protein is highly expressed in the RGC layer but it is also found at comparable levels in the photoreceptor, inner and outer plexiform retinal layers.^165^ ^166^ In the human optic nerve, Opa1 was detected along the axonal tracts both in the pre- and post-lamina cribosa regions.^167^ ^168^ The Opa1 protein is ubiquitous and abundant levels have been identified in non-ocular tissues such as the inner ear and various areas of the human brain, with a similar distribution pattern of the different isoforms.^169^ ^170^ Overall, these immunohistochemical studies indicate that differential tissue expression of the *OPA1* gene or its isoforms do not seem to underlie the selective vulnerability of RGCs in DOA.

### Protein function

The Opa1 protein is part of the large, dynamin GTPase family of mechanoenzymes and it was first identified in a screen for nuclear genes required for mtDNA maintenance in the budding yeast *Saccharomyces cerevisiae*. Both the human and yeast (Mgm1+) homologues show a high degree of evolutionary conservation and functional studies in DOA have revealed several other important cellular roles in addition to mtDNA maintenance.^171^ ^172^

#### Mitochondrial maintenance

Opa1 is an important pro-fusion protein and works in tandem with other members of the dynamin related mitofusin family (mfn-1 and mfn-2) to balance the pro-fission effects of other GTPases such as Drp1 and Fis-1.^173^ ^174^ It is therefore not surprising that mitochondrial network disruption is a key pathological feature seen in fibroblasts from DOA patients and other tissue cultures, including RGCs, where the expression of the Opa1 protein has been disrupted—for example, by small interfering RNAs.^162^ ^170^ ^175^ ^176^ Instead of a typical elongated, filamentous mitochondrial network, the latter becomes highly fragmented, with isolated mitochondria showing aberrant balloon-like enlargements. Transmission electron microscopy (TEM) also confirms altered mitochondrial ultrastructure with abnormal mitochondrial cristae organisation and paracrystalline inclusion bodies.^162^

Fusion is postulated to subserve a protective biological function by allowing the exchange and complementation of mitochondrial contents.^177^ ^178^ In this respect, neuronal cells with deficient mitochondrial fusion show a loss of mtDNA nucleoids and this important finding provides a possible disease mechanism, with the reduced expression of essential, mtDNA encoded, respiratory chain subunits resulting in a bioenergetic deficit, increased ROS levels and a greater susceptibility to undergo apoptosis.^179^ ^180^ These deleterious consequences could also contribute to the formation and clonal expansion of secondary mtDNA abnormalities such as mtDNA deletions, which have recently been identified in a subgroup of DOA families with a more complex multi-system involvement in addition to the optic neuropathy.^162^^–^164

#### Oxidative phosphorylation

Impaired mitochondrial biogenesis is central to the pathophysiology in DOA and there is good experimental evidence to support a predominant complex I defect. There is reduced mitochondrial membrane potential and ATP synthesis in fibroblast cultures carrying pathogenic *OPA1* mutations,^181^ ^182^ and in vivo disturbance of oxidative metabolism was evident in the calf muscle of patients with DOA using 31P-MRS.^183^ Immunoprecipitation studies also suggest that the Opa1 protein, in conjunction with other structural proteins such as the apoptosis inducing factor (AIF), interacts directly with complexes I, II and III and plays an important role in the assembly and stabilisation of their various component subunits.^176^ This provides another causal link between *OPA1* mutations and the resulting mitochondrial respiratory chain defect in DOA.

#### Apoptosis

Apoptosis is the final common pathway leading to RGC loss in DOA and cell death is likely be complex, being triggered by a combination of several interacting factors. Opa1 is processed by various, inner membrane proteases which include the presenilin associated rhomboid-like protein (Parl) and paraplegin, and this proteolytic cleavage results in a soluble, intermembrane form in addition to the integral, membrane bound form.^184^^–^186 These two proteins combine into oligomers which modulate the morphology of the inner mitochondrial membrane and the tightness of the cristae junctions, a process independent of the role of Opa1 in controlling fusion.^187^ Downregulation of Opa1 leads to aberrant cristae remodelling and the release of cytochrome *c* which is normally sequestered in the narrow junctions within the cristae.^175^ ^188^ This will either be sufficient on its own to induce the apoptotic cascade or will sensitise the cell to other pro-apoptotic stimuli such as AIF, increased ROS or the dissipation of the mitochondrial membrane potential.

### Animal models

There are now two established mouse models of DOA, with heterozygous mutations in exon 8 (c.1051C>T) and intron 10 (c.1065+5g>a) of the *OPA1* gene.^189^ ^190^ These two mutations are truncative, resulting in a 50% reduction in the expression of the Opa1 protein, and therefore represent a haploinsufficiency disease mechanism. In both models, homozygous mutant mice (*OPA1*−/−) died in utero during embryogenesis, highlighting the central role played by the Opa1 protein in early development. Heterozygous *OPA1*+/− mice faithfully replicated the human phenotype exhibiting a slowly progressive optic neuropathy and demonstrating objective reduction in visual function on psychophysical testing. There was a gradual loss of RGCs, leading to thinning of the retinal nerve fibre layer, and the surviving optic nerve axons had an abnormal morphology with swelling, distorted shapes, irregular areas of demyelination and myelin aggregates. Mitochondria within these axons showed disorganised cristae structures on TEM and cultured fibroblasts showed fragmentation of the mitochondrial network. These two *OPA1* mouse models represent powerful tools for dissecting the pathways mediating the preferential loss of RGCs in DOA, by allowing functional studies to be performed directly on these specialised cells, something which is not possible in humans given the lack of ocular tissues. These mutant mice will also prove useful when investigating the potential therapeutic benefit of future biological agents which could be injected into the vitreous cavity, allowing direct access with the RGC layer.

### Expanding phenotype

The hallmark of DOA is bilateral visual failure, but sensorineural deafness is a well reported association which is more commonly observed with some pathogenic mutations such as the p.R445H mutation.^191^^–^193 In his original description, Kjer also documented neurodevelopmental abnormalities in 10% of his Dutch cohort, although this has not been reported in other populations.^120^ ^125^ More recently, DOA families have been described where the optic atrophy was segregating with additional ocular and extraocular features such as progressive external ophthalmoplegia, ptosis, myopathy, ataxia, neuropathy, and an MS-like disorder.^162^^–^164 194 These syndromal variants of DOA, so-called “DOA plus”, have been linked with the accumulation of multiple mtDNA deletions, a finding consistent with the presence of cytochrome *c* oxidase (COX) deficient fibres in limb muscle biopsies from affected individuals.^195^ All of the causative *OPA1* mutations in these families were missense mutations with most, but not all of them, within the catalytic GTPase site of the protein. Although the actual proportion of families with these “DOA plus” phenotypes is as yet unknown, clinicians need to be aware of these additional clinical features as these can be subtle and therefore easily missed if not looked for specifically.

### Genetic counselling

There is currently no treatment to influence the disease process in DOA and clinical management, as for LHON, is supportive. Despite DOA being an autosomal dominant Mendelian disorder, genetic counselling for mutational carriers is difficult because of the pronounced inter- and intra-familial variability in the visual phenotype. There are no definite genotype–phenotype correlations but missense mutations within the GTPase protein domain are more likely to result in a complex, multi-systemic involvement, although it must be stressed that this observation requires further investigation in a larger cohort of DOA families.

With the availability of molecular testing for *OPA1* becoming more accessible, an increasing number of individuals with pathogenic mutations are being identified who are otherwise visually unaffected. The penetrance is >80% in well characterised, multi-generational families but figures as low as 43% have been reported, probably reflecting the different assessment criteria used (range 43–100%).^132^ ^196^ ^197^ This incomplete penetrance together with the variable clinical expressivity in both pure DOA and “DOA plus” families clearly imply that other, as yet unidentified, secondary factors are potentiating the deleterious effects of the *OPA1* mutations.

## MITOCHONDRIAL OPTIC NEUROPATHIES

The concept of inherited mitochondrial optic neuropathies is expanding with evidence of impaired mitochondrial function in other genetic diseases where optic nerve dysfunction is a recognised clinical feature (table 5). These include: (1) Friedreich’s ataxia where up to a third of cases have an optic neuropathy^198^ ^199^; (2) hereditary motor and sensory neuropathy type 6 (HMSN-6), a variant of Charcot–Marie–Tooth (CMT) disease defined by the presence of both optic atrophy and peripheral neuropathy^200^ ^201^; and (3) the hereditary spastic paraplegias (HSP).^202^^–^204

**Table 5:** Other inherited optic neuropathies linked to mitochondrial dysfunction

Disease	OMIM	Inheritance	Gene (protein)	Protein function	References
Friedreich’s ataxia	229300	Ar	*FXN* (frataxin)	Component of iron-sulfur clusters: regulation of mitochondrial respiratory chain activity and anti-oxidant properties	253, 254
HMSN-6	601152	Ad	*MFN2* (mitofusin-2)	Mitochondrial outer membrane GTPase: pro-fusion protein involved in maintenance of the mitochondrial network and mtDNA nucleoids (cf Opa1)	216, 217, 255
HSP-7	607259	Ar	*SPG7* (paraplegin)	Mitochondrial inner membrane protease: cleavage of Opa-1, control of mitochondrial ribosomal assembly and degradation of misfolded proteins	219, 256

Ar, autosomal recessive; Ad, autosomal dominant.

Glaucoma is the second most common cause of blindness in developed countries and accounts for about 10% of all blind registration in the UK.^205^ It is a primary, acquired optic neuropathy with a strong genetic component and *OPA1* mutations have been identified in a number of patients initially diagnosed with normal tension glaucoma, highlighting the similarities in optic disc features shared with DOA.^206^ ^207^ It is of note therefore that some studies have shown an association between the risk of developing glaucoma and certain *OPA1* polymorphic variants,^208^^–^210 with other investigators reporting mtDNA abnormalities in their glaucoma cohorts, such as an increased mtDNA copy number and reduced respiratory chain activities in peripheral blood lymphocytes.^211^ Although further studies are needed, these findings suggest a possible mitochondrial influence on the pathogenesis of glaucoma.

## UNIFYING HYPOTHESIS

The common theme in the various optic neuropathies described in this review is the vulnerability of RGCs to mitochondrial dysfunction. Although there is a high level of mitochondrial enzyme activity in RGCs,^212^ this phenomenon cannot be explained by a simple energetic deficit since photoreceptors have a much higher oxidative demand than RGCs and other mitochondrial disorders characterised by more severe complex I defects do not universally cause optic atrophy. It is possible that RGCs are preferentially involved because they are more sensitive to subtle imbalances in cellular redox state or increased ROS levels, but an attractive hypothesis implicates the differential mitochondrial concentration observed at the lamina cribosa.^213^ The lamina cribosa is a perforated collagen plate that marks the anatomical transition from the unmyelinated (pre-laminar) to the myelinated (post-laminar) segment of the human optic nerve. The pre-laminar section has a much higher concentration of mitochondria to support the higher energy demands of unmyelinated nerve conduction and it is likely that active processes involving the cytoskeletal architecture are needed to maintain this sharp mitochondrial gradient.^214^ ^215^ Pathological mechanisms which disrupt this unique structural feature would lead to impaired axonal transport, as seen in CMT^179^ ^216^ ^217^ and HSP,^218^ ^219^ and set up a vicious circle with fragmentation of the mitochondrial network at the lamina cribosa exacerbating even subtle mitochondrial energy deficits and eventually precipitating apoptotic cell death.

## CONCLUSION

LHON and DOA show an intriguing degree of clinical and mechanistic overlap, with both disorders caused by the selective degeneration of the RGC layer. They are the two most common inherited optic neuropathies and they provide strong evidence that the maintenance of RGCs is heavily dependent upon normal mitochondrial function. This is further substantiated by recent studies pointing towards a mitochondrial link in sporadic glaucoma and other genetic disorders where optic nerve dysfunction is a prominent clinical feature. Although major advances have been achieved in the two decades since the primary LHON mutations were identified, several key questions remain unanswered. What secondary factors account for the notable incomplete penetrance and male bias in LHON? What explains the variable disease expression in DOA, and why is there no gender bias in this disorder, given the similarity to LHON? What are the causative nuclear genes in *OPA1*-negative families and will they also involve mitochondrial dysfunction? What mechanisms underpin the preferential loss of RGCs in these mitochondrial optic neuropathies? The characterisation of recently developed animal models and future genetic and functional studies will hopefully reveal important pathophysiological pathways amenable to therapeutic interventions.

## References

[1] Schaefer AM McFarland R Blakely EL He L Whittaker RG Taylor RW Chinnery PF Turnbull DM Prevalence of mitochondrial DNA disease in adults. Ann Neurol 2008;63:35–9 17886296 10.1002/ana.21217

[2] Elliott HR Samuels DC Eden JA Relton CL Chinnery PF Pathogenic mitochondrial DNA mutations are common in the general population. Am J Hum Genet 2008;83:254–60 18674747 10.1016/j.ajhg.2008.07.004 PMC2495064

[3] Leber T Ueber hereditaere und congenital angelegte sehnervenleiden. Graefes Arch Opthal 1871;17:249–91

[4] Bell J Hereditary optic atrophy (Leber’s disease). Pearson K, ed. The treasury of human inheritance Cambridge: Cambridge University Press, 1931:345–423

[5] Imai Y Moriwaki D A probable case of cytoplasmic inheritance in man: a critique of Leber’s disease. J Genet 1936;33:163–7

[6] Lundsgaard R A genealogic, genetic and clinical study of 101 cases of retrobulbar optic neuritis in 20 Danish families. Acta Ophthalmol 1944;21:1–306

[7] Wallace DC Singh G Lott MT Hodge JA Schurr TG Lezza AM Elsas LJd Nikoskelainen EK Mitochondrial DNA mutation associated with Leber’s hereditary optic neuropathy. Science 1988;242:1427–30 3201231 10.1126/science.3201231

[8] Mackey DA Oostra RJ Rosenberg T Nikoskelainen E Bronte-Stewart J Poulton J Harding AE Govan G Bolhuis PA Norby S Primary pathogenic mtDNA mutations in multigeneration pedigrees with Leber hereditary optic neuropathy. Am J Hum Genet 1996;59:481–5 8755941 PMC1914749

[9] Mashima Y Yamada K Wakakura M Kigasawa K Kudoh J Shimizu N Oguchi Y Spectrum of pathogenic mitochondrial DNA mutations and clinical features in Japanese families with Leber’s hereditary optic neuropathy. Curr Eye Res 1998;17:403–8 9561832 10.1080/02713689808951221

[10] Yen MY Wang AG Chang WL Hsu WM Liu JH Wei YH Leber’s hereditary optic neuropathy-the spectrum of mitochondrial DNA mutations in Chinese patients. Jpn J Ophthalmol 2002;46:45–51 11853713 10.1016/s0021-5155(01)00460-9

[11] Macmillan C Kirkham T Fu K Allison V Andermann E Chitayat D Fortier D Gans M Hare H Quercia N Zackon D Shoubridge EA Pedigree analysis of French Canadian families with T14484C Leber’s hereditary optic neuropathy. Neurology 1998;50:417–22 9484365 10.1212/wnl.50.2.417

[12] Macmillan C Johns TA Fu K Shoubridge EA Predominance of the T14484C mutation in French-Canadian families with Leber hereditary optic neuropathy is due to a founder effect [letter]. Am J Hum Genet 2000;66:332–5 10631164 10.1086/302716 PMC1288340

[13] Taylor RW Jobling MS Turnbull DM Chinnery PF Frequency of rare mitochondrial DNA mutations in patients with suspected Leber’s hereditary optic neuropathy. J Med Genet 2003;40:e85 12843334 10.1136/jmg.40.7.e85 PMC1735533

[14] Man PY Griffiths PG Brown DT Howell N Turnbull DM Chinnery PF The epidemiology of Leber hereditary optic neuropathy in the North East of England. Am J Hum Genet 2003;72:333–9 12518276 10.1086/346066 PMC379226

[15] Spruijt L Kolbach DN de Coo RF Plomp AS Bauer NJ Smeets HJ de Die-Smulders CEM Influence of mutation type on clinical expression of Leber hereditary optic neuropathy. Am J Ophthalmol 2006;141:676–82 16564802 10.1016/j.ajo.2005.11.007

[16] Puomila A Hamalainen P Kivioja S Savontaus ML Koivumaki S Huoponen K Nikoskelainen E Epidemiology and penetrance of Leber hereditary optic neuropathy in Finland. Eur J Hum Genet 2007;15:1079–89 17406640 10.1038/sj.ejhg.5201828

[17] Mackey DA Buttery RG Leber hereditary optic neuropathy in Australia. Aust N Z J Ophthalmol 1992;20:177–84 1449769 10.1111/j.1442-9071.1992.tb00937.x

[18] Yu-Wai-Man P Bateman DE Hudson G Griffiths PG Chinnery PF Leber hereditary optic neuropathy presenting in a 75-year-old man. J Neuroophthalmol 2008;28:155 18562849 10.1097/WNO.0b013e3181772db4

[19] Dagi LR Rizzo JF 3rd Cestari DM Leber hereditary optic neuropathy in an octogenarian. J Neuroophthalmol 2008;28:156 18562851 10.1097/WNO.0b013e3181772320

[20] Harding AE Sweeney MG Govan GG Riordan-Eva P Pedigree analysis in Leber hereditary optic neuropathy families with a pathogenic mtDNA mutation. Am J Hum Genet 1995;57:77–86 7611298 PMC1801226

[21] Newman NJ Lott MT Wallace DC The clinical characteristics of pedigrees of Leber’s hereditary optic neuropathy with the 11778 mutation. Am J Ophthalmol 1991;111:750–62 2039048 10.1016/s0002-9394(14)76784-4

[22] Johns DR Smith KH Miller NR Leber’s hereditary optic neuropathy. Clinical manifestations of the 3460 mutation. Arch Ophthalmol 1992;110:1577–81 1444915 10.1001/archopht.1992.01080230077025

[23] Johns DR Heher KL Miller NR Smith KH Leber’s hereditary optic neuropathy. Clinical manifestations of the 14484 mutation. Arch Ophthalmol 1993;111:495–8 8470982 10.1001/archopht.1993.01090040087038

[24] Biousse V Brown MD Newman NJ Allen JC Rosenfeld J Meola G Wallace DC De novo 14484 mitochondrial DNA mutation in monozygotic twins discordant for Leber’s hereditary optic neuropathy. Neurology 1997;49:1136–8 9339703 10.1212/wnl.49.4.1136

[25] Savini G Barboni P Valentino ML Montagna P Cortelli P De Negri AM Sadun F Bianchi S Longanesi L Zanini M Carelli V Retinal nerve fiber layer evaluation by optical coherence tomography in unaffected carriers with Leber’s hereditary optic neuropathy mutations. Ophthalmology 2005;112:127–31 15629832 10.1016/j.ophtha.2004.09.033

[26] Quiros PA Torres RJ Salomao S Berezovsky A Carelli V Sherman J Sadun F De Negri A Belfort R Sadun AA Colour vision defects in asymptomatic carriers of the Leber’s hereditary optic neuropathy (LHON) mtDNA 11778 mutation from a large Brazilian LHON pedigree: a case-control study. Br J Ophthalmol 2006;90:150–3 16424523 10.1136/bjo.2005.074526 PMC1860163

[27] Sadun AA Salomao SR Berezovsky A Sadun F Denegri AM Quiros PA Chicani F Ventura D Barboni P Sherman J Sutter E Belfort R Jr Carelli V Patsi J Kervinen M Finel M Hassinen IE Subclinical carriers and conversions in Leber hereditary optic neuropathy: a prospective psychophysical study. Trans Am Ophthalmol Soc 2006;104:51–61 17471325 PMC1809912

[28] Nikoskelainen EK Huoponen K Juvonen V Lamminen T Nummelin K Savontaus ML Ophthalmologic findings in Leber hereditary optic neuropathy, with special reference to mtDNA mutations. Ophthalmology 1996;103:504–14 8600429 10.1016/s0161-6420(96)30665-9

[29] Sugisaka E Ohde H Shinoda K Mashima Y Woman with atypical unilateral Leber’s hereditary optic neuropathy with visual improvement. Clin Exp Ophthalmol 2007;35:868–70 10.1111/j.1442-9071.2007.01628.x 18173420

[30] Nikoskelainen EK Clinical picture of LHON. Clin Neurosci 1994;2:115–20

[31] Riordan-Eva P Harding AE Leber’s hereditary optic neuropathy: the clinical relevance of different mitochondrial DNA mutations. J Med Genet 1995;32:81–7 7760326 10.1136/jmg.32.2.81 PMC1050224

[32] Mackey D Howell N A variant of Leber hereditary optic neuropathy characterized by recovery of vision and by an unusual mitochondrial genetic etiology. Am J Hum Genet 1992;51:1218–28 1463007 PMC1682921

[33] Stone EM Newman NJ Miller NR Johns DR Lott MT Wallace DC Visual recovery in patients with Leber’s hereditary optic neuropathy and the 11778 mutation. J Clin Neuroophthalmol 1992;12:10–4 1532593

[34] Barboni P Savini G Valentino ML La Morgia C Bellusci C De Negri AM Sadun F Carta A Carbonelli M Sadun AA Carelli V Leber’s hereditary optic neuropathy with childhood onset. Invest Ophthalmol Vis Sci 2006;47:5303–9 17122117 10.1167/iovs.06-0520

[35] Bower SP Hawley I Mackey DA Cardiac arrhythmia and Leber’s hereditary optic neuropathy [letter]. Lancet 1992;339:1427–8 1350847 10.1016/0140-6736(92)91257-9

[36] Nikoskelainen EK Savontaus ML Huoponen K Antila K Hartiala J Pre-excitation syndrome in Leber’s hereditary optic neuropathy. Lancet 1994;344:857–8 7916404 10.1016/s0140-6736(94)92830-4

[37] Nikoskelainen EK Marttila RJ Huoponen K Juvonen V Lamminen T Sonninen P Savontaus ML Leber’s “plus”: neurological abnormalities in patients with Leber’s hereditary optic neuropathy. J Neurol Neurosurg Psychiatry 1995;59:160–4 7629530 10.1136/jnnp.59.2.160 PMC485991

[38] Meire FM Van Coster R Cochaux P Obermaier-Kusser B Candaele C Martin JJ Neurological disorders in members of families with Leber’s hereditary optic neuropathy (LHON) caused by different mitochondrial mutations. Ophthalm Genet 1995;16:119–26 10.3109/13816819509059971 8556281

[39] Mashima Y Kigasawa K Hasegawa H Tani M Oguchi Y High incidence of pre-excitation syndrome in Japanese families with Leber’s hereditary optic neuropathy. Clin Genet 1996;50:535–7 9147893 10.1111/j.1399-0004.1996.tb02732.x

[40] De Vries DD Went LN Bruyn GW Scholte HR Hofstra RM Bolhuis PA van Oost BA Genetic and biochemical impairment of mitochondrial complex I activity in a family with Leber hereditary optic neuropathy and hereditary spastic dystonia. Am J Hum Genet 1996;58:703–11 8644732 PMC1914692

[41] Howell N Kubacka I Xu M McCullough DA Leber hereditary optic neuropathy: involvement of the mitochondrial ND1 gene and evidence for an intragenic suppressor mutation. Am J Hum Genet 1991;48:935–42 2018041 PMC1683051

[42] Jun AS Brown MD Wallace DC A mitochondrial DNA mutation at nucleotide pair 14459 of the NADH dehydrogenase subunit 6 gene associated with maternally inherited Leber hereditary optic neuropathy and dystonia. Proc Natl Acad Sci USA 1994;91:6206–10 8016139 10.1073/pnas.91.13.6206 PMC44167

[43] Gropman A Chen TJ Perng CL Krasnewich D Chernoff E Tifft C Wong LJ Variable clinical manifestation of homoplasmic G14459A mitochondrial DNA mutation. Am J Med Genet Part A 2004;124:377–82 10.1002/ajmg.a.20456 14735585

[44] Tarnopolsky MA Baker SK Myint T Maxner CE Robitaille J Robinson BH Clinical variability in maternally inherited Leber hereditary optic neuropathy with the G14459A mutation. Am J Med Genet Part A 2004;124:372–6 10.1002/ajmg.a.20449 14735584

[45] Blakely EL de Silva R King A Schwarzer V Harrower T Dawidek G Turnbull DM Taylor RW LHON/MELAS overlap syndrome associated with a mitochondrial MTND1 gene mutation. Eur J Hum Genet 2005;13:623–7 15657614 10.1038/sj.ejhg.5201363

[46] Spruijt L Smeets HJ Hendrickx A Bettink-Remeijer MW Maat-Kievit A Schoonderwoerd KC Sluiter W de Coo IF Hintzen RQ A MELAS-associated ND1 mutation causing Leber hereditary optic neuropathy and spastic dystonia. Arch Neurol 2007;64:890–3 17562939 10.1001/archneur.64.6.890

[47] Harding AE Sweeney MG Miller DH Mumford CJ Kellar-Wood H Menard D McDonald WI Compston DA Occurrence of a multiple sclerosis-like illness in women who have a Leber’s hereditary optic neuropathy mitochondrial DNA mutation. Brain 1992;115:979–89 1393514 10.1093/brain/115.4.979

[48] Kellar-Wood H Robertson N Govan GG Compston DA Harding AE Leber’s hereditary optic neuropathy mitochondrial DNA mutations in multiple sclerosis. Ann Neurol 1994;36:109–12 8024249 10.1002/ana.410360121

[49] Jansen PH van der Knaap MS de Coo IF Leber’s hereditary optic neuropathy with the 11 778 mtDNA mutation and white matter disease resembling multiple sclerosis: clinical, MRI and MRS findings. J Neurol Sci 1996;135:176–80 8867076 10.1016/0022-510x(95)00287-c

[50] Vanopdenbosch L Dubois B D’Hooghe MB Meire F Carton H Mitochondrial mutations of Leber’s hereditary optic neuropathy: a risk factor for multiple sclerosis. J Neurol 2000;247:535–43 10993496 10.1007/s004150070153

[51] Govan GG Smith PR Kellar-Wood H Schapira AH Harding AE HLA class II genotypes in Leber’s hereditary optic neuropathy. J Neurol Sci 1994;126:193–6 7853025 10.1016/0022-510x(94)90272-0

[52] Smith PR Cooper JM Govan GG Riordan-Eva P Harding AE Schapira AH Antibodies to human optic nerve in Leber’s hereditary optic neuropathy. J Neurol Sci 1995;130:134–8 8586976 10.1016/0022-510x(95)00021-s

[53] Chalmers RM Govan GG Schapira AH Harding AE HLA class I genotypes in Leber’s hereditary optic neuropathy. J Neurol Sci 1996;135:173–5 8867075 10.1016/0022-510x(95)00286-b

[54] Sapey E Burdon MA Nightingale S Evidence of active demyelination in a man with Leber’s hereditary optic neuropathy mtDNA 14484 genotype. Neuro-Ophthalmology 2001;26:119–26

[55] Kovacs GG Hoftberger R Majtenyi K Horvath R Barsi P Komoly S Lassmann H Budka H Jakab G Neuropathology of white matter disease in Leber’s hereditary optic neuropathy. Brain 2005;128:35–41 15483043 10.1093/brain/awh310

[56] Sherman J Kleiner L Visual-system dysfunction in Lebers hereditary optic neuropathy. Clin Neurosci 1994;2:121–9

[57] Smith JL Tse DT Byrne SF Johns DR Stone EM Optic nerve sheath distention in Leber’s optic neuropathy and the significance of the “Wallace mutation”. J Clin Neuroophthalmol 1990;10:231–8 2150839

[58] de Gottrau P Buchi ER Daicker B Distended optic nerve sheaths in Leber’s hereditary optic neuropathy. J Clin Neuroophthalmol 1992;12:89–93 1629376

[59] Dotti MT Caputo N Signorini E Federico A Magnetic resonance imaging findings in Leber’s hereditary optic neuropathy. Eur Neurol 1992;32:17–9 1563448 10.1159/000116781

[60] Mashima Y Oshitari K Imamura Y Momoshima S Shiga H Oguchi Y Orbital high resolution magnetic resonance imaging with fast spin echo in the acute stage of Leber’s hereditary optic neuropathy. J Neurol Neurosurg Psychiatry 1998;64:124–7 9436742 10.1136/jnnp.64.1.124 PMC2169910

[61] Vaphiades MS Newman NJ Optic nerve enhancement on orbital magnetic resonance imaging in Leber’s hereditary optic neuropathy. J Neuroophthalmol 1999;19:238–9 10608675

[62] Inglese M Rovaris M Bianchi S Mancardi GL Ghezzi A Salvi F Cortelli P Filippi M MRI, MTI, and DWI study of the optic nerve, brain, and cervical cord from patients with Leber hereditary optic neuropathy. Neurology 2000;54:A320–A

[63] Barbiroli B Montagna P Cortelli P Iotti S Lodi R Barboni P Monari L Lugaresi E Frassineti C Zaniol P Defective brain and muscle energy metabolism shown by in vivo 31P magnetic resonance spectroscopy in nonaffected carriers of 11778 mtDNA mutation. Neurology 1995;45:1364–9 7617199 10.1212/wnl.45.7.1364

[64] Cortelli P Montagna P Pierangeli G Lodi R Barboni P Liguori R Carelli V Iotti S Zaniol P Lugaresi E Barbiroli B Clinical and brain bioenergetics improvement with idebenone in a patient with Leber’s hereditary optic neuropathy: a clinical and 31P-MRS study. J Neurol Sci 1997;148:25–31 9125387 10.1016/s0022-510x(96)00311-5

[65] Lodi R Taylor DJ Tabrizi SJ Kumar S Sweeney M Wood NW Styles P Radda GK Schapira AHV In vivo skeletal muscle mitochondrial function in Leber’s hereditary optic neuropathy assessed by P-31 magnetic resonance spectroscopy. Ann Neurol 1997;42:573–9 9382468 10.1002/ana.410420407

[66] Lodi R Montagna P Cortelli P Iotti S Cevoli S Carelli V Barbiroli B ‘Secondary’ 4216/ND1 and 13708/ND5 Leber’s hereditary optic neuropathy mitochondrial DNA mutations do not further impair in vivo mitochondrial oxidative metabolism when associated with the 11778/ND4 mitochondrial DNA mutation. Brain 2000;123:1896–902 10960053 10.1093/brain/123.9.1896

[67] Lodi R Carelli V Cortelli P Iotti S Valentino ML Barboni P Pallotti F Montagna P Barbiroli B Phosphorus MR spectroscopy shows a tissue specific in vivo distribution of biochemical expression of the G3460A mutation in Leber’s hereditary optic neuropathy. J Neuro Neurosurg Psychiatry 2002;72:805–7 10.1136/jnnp.72.6.805 PMC1737903 12023431

[68] Beretta S Mattavelli L Sala G Tremolizzo L Schapira AH Martinuzzi A Carelli V Ferrarese C Leber hereditary optic neuropathy mtDNA mutations disrupt glutamate transport in cybrid cell lines. Brain 2004;127:2183–92 15342361 10.1093/brain/awh258

[69] Beretta S Wood JPM Derham B Sala G Tremolizzo L Ferrarese C Osborne NN Partial mitochondrial complex I inhibition induces oxidative damage and perturbs glutamate transport in primary retinal cultures. Relevance to Leber hereditary optic neuropathy (LHON). Neurobiol Dis 2006;24:308–17 16959493 10.1016/j.nbd.2006.07.016

[70] Floreani M Napoli E Martinuzzi A Pantano G De Riva V Trevisan R Bisetto E Valente L Carelli V Dabbeni-Sala F Antioxidant defences in cybrids harboring mtDNA mutations associated with Leber’s hereditary optic neuropathy. Febs J 2005;272:1124–35 15720387 10.1111/j.1742-4658.2004.04542.x

[71] Carelli V Rugolo M Sgarbi G Ghelli A Zanna C Baracca A Lenaz G Napoli E Martinuzzi A Solaini G Bioenergetics shapes cellular death pathways in Leber’s hereditary optic neuropathy: a model of mitochondrial neurodegeneration. Biochim Biophys Acta Bioenerg 2004;1658:172–9 10.1016/j.bbabio.2004.05.009 15282189

[72] Danielson SR Wong A Carelli V Martinuzzi A Schapira AH Cortopassi GA Cells bearing mutations causing Leber’s hereditary optic neuropathy are sensitized to Fas-induced apoptosis. J Biol Chem 2002;277:5810–5 11741983 10.1074/jbc.M110119200

[73] Zanna C Ghelli A Porcelli AM Martinuzzi A Carelli V Rugolo M Caspase-independent death of Leber’s hereditary optic neuropathy cybrids is driven by energetic failure and mediated by AIF and endonuclease G. Apoptosis 2005;10:997–1007 16151635 10.1007/s10495-005-0742-5

[74] Klivenyi P Karg E Rozsa C Horvath R Komoly S Nemeth I Turi S Vecsei L alpha-Tocopherol/lipid ratio in blood is decreased in patients with Leber’s hereditary optic neuropathy and asymptomatic carriers of the 11778 mtDNA mutation. J Neurol Neurosurg Psychiatry 2001;70:359–62 11181859 10.1136/jnnp.70.3.359 PMC1737282

[75] Yen MY Kao SH Wang AG Wei YH Increased 8-hydroxy-2’-deoxyguanosine in leukocyte DNA in Leber’s hereditary optic neuropathy. Invest Ophthalmol Vis Sci 2004;45:1688–91 15161827 10.1167/iovs.03-0568

[76] Zhang X Jones D Gonzalez-Lima F Mouse model of optic neuropathy caused by mitochondrial complex I dysfunction. Neurosci Lett 2002;326:97–100 12057837 10.1016/s0304-3940(02)00327-0

[77] Qi XP Lewin AS Hauswirth WW Guy J Suppression of complex I gene expression induces optic neuropathy. Ann Neurol 2003;53:198–205 12557286 10.1002/ana.10426

[78] Qi XP Sun L Lewin AS Hauswirth WW Guy J The mutant human ND4 subunit of complex I induces optic neuropathy in the mouse. Invest Ophthalmol Vis Sci 2007;48:1–10 17197509 10.1167/iovs.06-0789

[79] Smith KH Johns DR Heher KL Miller NR Heteroplasmy in Leber’s hereditary optic neuropathy. Arch Ophthalmol 1993;111:1486–90 8240102 10.1001/archopht.1993.01090110052022

[80] Chinnery PF Andrews RM Turnbull DM Howell NN Leber hereditary optic neuropathy: does heteroplasmy influence the inheritance and expression of the G11778A mitochondrial DNA mutation? Am J Med Genet 2001;98:235–43 11169561 10.1002/1096-8628(20010122)98:3<235::aid-ajmg1086>3.0.co;2-o

[81] Brown WM George M Jr Wilson AC Rapid evolution of animal mitochondrial DNA. Proc Natl Acad Sci USA 1979;76:1967–71 109836 10.1073/pnas.76.4.1967 PMC383514

[82] Wallace DC Brown MD Lott MT Mitochondrial DNA variation in human evolution and disease. Gene 1999;238:211–30 10570998 10.1016/s0378-1119(99)00295-4

[83] Torroni A Huoponen K Francalacci P Petrozzi M Morelli L Scozzari R Obinu D Savontaus ML Wallace DC Classification of European mtDNAs from an analysis of three European populations. Genetics 1996;144:1835–50 8978068 10.1093/genetics/144.4.1835 PMC1207732

[84] Hofmann S Jaksch M Bezold R Mertens S Aholt S Paprotta A Gerbitz KD Population genetics and disease susceptibility: characterization of central European haplogroups by mtDNA gene mutations, correlation with D loop variants and association with disease. Hum Mol Genet 1997;6:1835–46 9302261 10.1093/hmg/6.11.1835

[85] Hudson G Carelli V Spruijt L Gerards M Mowbray C Achilli A Pyle A Elson J Howell N La Morgia C Valentino ML Huoponen K Savontaus ML Nikoskelainen E Sadun AA Salomao SR Belfort R Griffiths P Man PYW de Coo RFM Horvath R Zeviani M Smeets HJT Torroni A Chinnery PF Clinical expression of Leber hereditary optic neuropathy is affected by the mitochondrial DNA-haplogroup background. Am J Hum Genet 2007;81:228–33 17668373 10.1086/519394 PMC1950812

[86] Dudkina NV Eubel H Keegstra W Boekema EJ Braun HP Structure of a mitochondrial supercomplex formed by respiratory-chain complexes I and III. Proc Natl Acad Sci USA 2005;102:3225–9 15713802 10.1073/pnas.0408870102 PMC552927

[87] Carelli V Achilli A Valentino ML Rengo C Semino O Pala M Olivieri A Mattiazzi M Pallotti F Carrara F Zeviani M Leuzzi V Carducci C Valle G Simionati B Mendieta L Salomao S Belfort R Jr Sadun AA Torroni A Haplogroup effects and recombination of mitochondrial DNA: novel clues from the analysis of Leber hereditary optic neuropathy pedigrees. Am J Hum Genet 2006;78:564–74 16532388 10.1086/501236 PMC1424694

[88] Vergani L Martinuzzi A Carelli V Cortelli P Montagna P Schievano G Carrozzo R Angelini C Lugaresi E MtDNA mutations associated with Leber’s hereditary optic neuropathy: studies on cytoplasmic hybrid (cybrid) cells. Biochem Biophys Res Commun 1995;210:880–8 7763260 10.1006/bbrc.1995.1740

[89] Tharaphan P Chuenkongkaew WL Luangtrakool K Sanpachudayan T Suktitipat B Suphavilai R Srisawat C Sura T Lertrit P Mitochondrial DNA haplogroup distribution in pedigrees of Southeast Asian G11778A Leber hereditary optic neuropathy. J Neuroophthalmol 2006;26:264–7 17204919 10.1097/01.wno.0000249318.88991.c4

[90] Bu XD Rotter JI X chromosome-linked and mitochondrial gene control of Leber hereditary optic neuropathy: evidence from segregation analysis for dependence on X chromosome inactivation. Proc Natl Acad Sci USA 1991;88:8198–202 1896469 10.1073/pnas.88.18.8198 PMC52474

[91] Bu X Rotter JI Leber hereditary optic neuropathy: estimation of number of embryonic precursor cells and disease threshold in heterozygous affected females at the X-linked locus. Clin Genet 1992;42:143–8 1395084 10.1111/j.1399-0004.1992.tb03226.x

[92] Nakamura M Fujiwara Y Yamamoto M The two locus control of Leber hereditary optic neuropathy and a high penetrance in Japanese pedigrees. Hum Genet 1993;91:339–41 8500789 10.1007/BF00217353

[93] Pegoraro E Carelli V Zeviani M Cortelli P Montagna P Barboni P Angelini C Hoffman EP X-inactivation patterns in female Leber’s hereditary optic neuropathy patients do not support a strong X-linked determinant. Am J Med Genet 1996;61:356–62 8834048 10.1002/(SICI)1096-8628(19960202)61:4<356::AID-AJMG10>3.0.CO;2-R

[94] Oostra RJ Kemp S Bolhuis PA Bleeker-Wagemakers EM No evidence for ‘skewed’ inactivation of the X-chromosome as cause of Leber’s hereditary optic neuropathy in female carriers. Hum Genet 1996;97:500–5 8834251 10.1007/BF02267075

[95] Hudson G Carelli V Horvath R Zeviani M Smeets HJ Chinnery PF X-Inactivation patterns in females harboring mtDNA mutations that cause Leber hereditary optic neuropathy. Mol Vis 2007;13:2339–43 18199976

[96] Chen JD Cox I Denton MJ Preliminary exclusion of an X-linked gene in Leber optic atrophy by linkage analysis. Hum Genet 1989;82:203–7 2731932 10.1007/BF00291154

[97] Carvalho MR Muller B Rotzer E Berninger T Kommerell G Blankenagel A Savontaus ML Meitinger T Lorenz B Leber’s hereditary optic neuroretinopathy and the X-chromosomal susceptibility factor: no linkage to DXs7. Hum Hered 1992;42 (5):316–20 1360941 10.1159/000154089

[98] Sweeney MG Davis MB Lashwood A Brockington M Toscano A Harding AE Evidence against an X-linked locus close to DXS7 determining visual loss susceptibility in British and Italian families with Leber hereditary optic neuropathy. Am J Hum Genet 1992;51:741–8 1415219 PMC1682819

[99] Handoko HY Wirapati PJ Sudoyo HA Sitepu M Marzuki S Meiotic breakpoint mapping of a proposed X linked visual loss susceptibility locus in Leber’s hereditary optic neuropathy. J Med Genet 1998;35:668–71 9719375 10.1136/jmg.35.8.668 PMC1051394

[100] Hudson G Keers S Man PYW Griffiths P Huoponen K Savontaus ML Nikoskelainen E Zeviani M Carrara F Horvath R Karcagi V Spruijt L de Coo IFM Smeets HJM Chinnery PF Identification of an X-chromosomal locus and haplotype modulating the phenotype of a mitochondrial DNA disorder. Am J Hum Genet 2005;77:1086–91 16380918 10.1086/498176 PMC1285165

[101] Shankar SP Fingert JH Carelli V Valentino ML King TM Daiger SP Salomao SR Berezovsky A Belfort R Jr Braun TA Sheffield VC Sadun AA Stone EM Evidence for a novel x-linked modifier locus for Leber hereditary optic neuropathy. Ophthalmic Genet 2008;29:17–24 18363168 10.1080/13816810701867607

[102] Nikoskelainen EK Savontaus ML Wanne OP Katila MJ Nummelin KU Leber’s hereditary optic neuroretinopathy, a maternally inherited disease. A genealogic study in four pedigrees. Arch Ophthalmol 1987;105:665–71 3619743 10.1001/archopht.1987.01060050083043

[103] Johns DR Smith KH Miller NR Sulewski ME Bias WB Identical twins who are discordant for Leber’s hereditary optic neuropathy. Arch Ophthalmol 1993;111:1491–4 8240103 10.1001/archopht.1993.01090110057023

[104] Lam BL Identical twins no longer discordant for Leber’s hereditary optic neuropathy [letter]. Arch Ophthalmol 1998;116:956–7 9682718

[105] Riordan-Eva P Sanders MD Govan GG Sweeney MG Da Costa J Harding AE The clinical features of Leber’s hereditary optic neuropathy defined by the presence of a pathogenic mitochondrial DNA mutation. Brain 1995;118:319–37 7735876 10.1093/brain/118.2.319

[106] Charlmers RM Harding AE A case-control study of Leber’s hereditary optic neuropathy. Brain 1996;119:1481–6 8931573 10.1093/brain/119.5.1481

[107] Tsao K Aitken PA Johns DR Smoking as an aetiological factor in a pedigree with Leber’s hereditary optic neuropathy. Br J Ophthalmol 1999;83:577–81 10216058 10.1136/bjo.83.5.577 PMC1723036

[108] Sadun AA Carelli V Salomao SR Berezovsky A Quiros PA Sadun F DeNegri AM Andrade R Moraes M Passos A Kjaer P Pereira J Valentino ML Schein S Belfort R Extensive investigation of a large Brazilian pedigree of 11778/haplogroup J Leber hereditary optic neuropathy. Am J Ophthalmol 2003;136:231–8 12888043 10.1016/s0002-9394(03)00099-0

[109] Kerrison JB Miller NR Hsu F Beaty TH Maumenee IH Smith KH Savino PJ Stone EM Newman NJ A case-control study of tobacco and alcohol consumption in Leber hereditary optic neuropathy. Am J Ophthalmol 2000;130:803–12 11124301 10.1016/s0002-9394(00)00603-6

[110] Mackey DA Fingert JH Luzhansky JZ McCluskey PJ Howell N Hall AJH Pierce AB Hoy JF Leber’s hereditary optic neuropathy triggered by antiretroviral therapy for human immunodeficiency virus. Eye 2003;17:312–7 12724691 10.1038/sj.eye.6700362

[111] Sanchez RN Smith AJ Carelli V Sadun AA Keltner JL Leber hereditary optic neuropathy possibly triggered by exposure to tire fire. J Neuroophthalmol 2006;26:268–72 17204920 10.1097/01.wno.0000249320.27110.ab

[112] Carelli V Franceschini F Venturi S Barboni P Savini G Barbieri G Pirro E La Morgia C Valentino ML Zanardi F Violante FS Mattioli S Grand rounds: could occupational exposure to n-hexane and other solvents precipitate visual failure in Leber hereditary optic neuropathy? Environ Health Perspect 2007;115:113–5 17366829 10.1289/ehp.9245 PMC1797843

[113] Howell N Mackey DA Low-penetrance branches in matrilineal pedigrees with Leber hereditary optic neuropathy. Am J Hum Genet 1998;63:1220–4 9867707 10.1086/302049 PMC1377511

[114] Sadun F De Negri AM Carelli V Salomao SR Berezovsky A Andrade R Moraes M Passos A Belfort R Da Rosa AB Quiros P Sadun AA Ophthalmologic findings in a large pedigree of 11778/Haplogroup J Leber hereditary optic neuropathy. Am J Ophthalmol 2004;137:271–7 14962416 10.1016/j.ajo.2003.08.010

[115] Mashima Y Kigasawa K Wakakura M Oguchi Y Do idebenone and vitamin therapy shorten the time to achieve visual recovery in Leber hereditary optic neuropathy? J Neuroophthalmol 2000;20:166–70 11001192 10.1097/00041327-200020030-00006

[116] Carelli V Valentino ML Liguori R Meletti S Vetrugno R Provini F Mancardi GL Bandini F Baruzzi A Montagna P Leber’s hereditary optic neuropathy (LHON/11778) with myoclonus: report of two cases. J Neurol Neurosurg Psychiatry 2001;71:813–6 11723211 10.1136/jnnp.71.6.813 PMC1737658

[117] Barnils N Mesa E Munoz S Ferrer-Artola A Arruga J Response to idebenone and multivitamin therapy in Leber’s hereditary optic neuropathy. Arch Soc Esp Oftalmol 2007;82:377–80 17573650 10.4321/s0365-66912007000600012

[118] Newman NJ Biousse V David R Bhatti MT Hamilton SR Farris BK Lesser RL Newman SA Turbin RE Chen K Keaney RP Prophylaxis for second eye involvement in Leber hereditary optic neuropathy: an open-labeled, nonrandomized multicenter trial of topical brimonidine purite. Am J Ophthalmol 2005;140:407–15 16083844 10.1016/j.ajo.2005.03.058

[119] Batten B A family suffering from hereditary optic atrophy. Transactions of the Ophthalmological Society UK 1896;16:125

[120] Kjer B Infantile optic atrophy with dominant transmission. Dan Med Bull 1956;3:135–41 13356616

[121] Lyle WM Genetic risks. A reference for eye care practitioners Waterloo, Ontario, Canada: University of Waterloo Press, 1990

[122] Thiselton DL Alexander C Morris A Brooks S Rosenberg T Eiberg H Kjer B Kjer P Bhattacharya SS Votruba M A frameshift mutation in exon 28 of the OPA1 gene explains the high prevalence of dominant optic atrophy in the Danish population: evidence for a founder effect. Hum Genet 2001;109:498–502 11735024 10.1007/s004390100600

[123] Kline LB Glaser JS Dominant optic atrophy – clinical profile. Arch Ophthalmol 1979;97:1680–6 314284 10.1001/archopht.1979.01020020248013

[124] Hoyt CS Autosomal dominant optic atrophy – a spectrum of disability. Ophthalmology 1980;87:245–51 7422264 10.1016/s0161-6420(80)35247-0

[125] Kjer P Infantile optic atrophy with dominant mode of inheritance: a clinical and genetic study of 19 Danish families. Acta Ophthalmol 1959;(Suppl 54):1–146 13660776

[126] Eliott D Traboulsi EI Maumenee IH Visual prognosis in autosomal dominant optic atrophy (Kjer type). Am J Ophthalmol 1993;115:360–7 8442497 10.1016/s0002-9394(14)73589-5

[127] Votruba M Moore AT Bhattacharya SS Clinical features, molecular genetics, and pathophysiology of dominant optic atrophy. J Med Genet 1998;35:793–800 9783700 10.1136/jmg.35.10.793 PMC1051452

[128] Puomila A Huoponen K Mantyjarvi M Hamalainen P Paananen R Sankila EM Savontaus ML Somer M Nikoskelainen E Dominant optic atrophy: correlation between clinical and molecular genetic studies. Acta Ophthalmol Scandinavica 2005;83:337–46 10.1111/j.1600-0420.2005.00448.x 15948788

[129] Cohn AC Toomes C Hewitt AW Kearns LS Inglehearn CF Craig JE Mackey DA The natural history of OPA1-related autosomal dominant optic atrophy. Br J Ophthalmol 2008;24:24 10.1136/bjo.2007.134726 18653586

[130] Kjer B Eiberg H Kjer P Rosenberg T Dominant optic atrophy mapped to chromosome 3q region. II. Clinical and epidemiological aspects. Acta Ophthalmol Scandinavica 1996;74:3–7 10.1111/j.1600-0420.1996.tb00672.x 8689476

[131] Votruba M Fitzke FW Holder GE Carter A Bhattacharya SS Moore AT Clinical features in affected individuals from 21 pedigrees with dominant optic atrophy. Arch Ophthalmol 1998;116:351–8 9514489 10.1001/archopht.116.3.351

[132] Cohn AC Toomes C Potter C Towns KV Hewitt AW Inglehearn CF Craig JE Mackey DA Autosomal dominant optic atrophy: penetrance and expressivity in patients with OPA1 mutations. Am J Ophthalmol 2007;143:656–62 17306754 10.1016/j.ajo.2006.12.038

[133] Berninger TA Jaeger W Krastel H Electrophysiology and color perimetry in dominant infantile optic atrophy. Br J Ophthalmol 1991;75:49–52 1991088 10.1136/bjo.75.1.49 PMC504107

[134] Bremner FD Tomlin EA Shallo-Hoffmann J Votruba M Smith SE The pupil in dominant optic atrophy. Invest Ophthalmol Vis Sci 2001;42:675–8 11222526

[135] Votruba M Thiselton D Bhattacharya SS Optic disc morphology of patients with OPA1 autosomal dominant optic atrophy. Br J Ophthalmol 2003;87:48–53 12488262 10.1136/bjo.87.1.48 PMC1771445

[136] Fournier AV Damji KF Epstein DL Pollock SC Disc excavation in dominant optic atrophy. Ophthalmology 2001;108:1595–602 11535456 10.1016/s0161-6420(01)00696-0

[137] Ito Y Nakamura M Yamakoshi T Lin J Yatsuya H Terasaki H Reduction of inner retinal thickness in patients with autosomal dominant optic atrophy associated with OPA1 mutations. Invest Ophthalmol Vis Sci 2007;48:4079–86 17724190 10.1167/iovs.07-0024

[138] Kim TW Hwang JM Stratus OCT in dominant optic atrophy: features differentiating it from glaucoma. J Glaucoma 2007;16:655–8 18091450 10.1097/IJG.0b013e31804d23aa

[139] Johnston PB Gaster RN Smith VC Tripathi RC Clinicopathologic study of autosomal dominant optic atrophy. Am J Ophthalmol 1979;88:868–75 315716 10.1016/0002-9394(79)90565-8

[140] Kjer P Jensen OA Klinken L Histopathology of eye, optic-nerve and brain in a case of dominant optic atrophy. Acta Ophthalmol 1983;61:300–12 6880639 10.1111/j.1755-3768.1983.tb01424.x

[141] Votruba M Leary S Losseff N Bhattacharya SS Moore AT Miller DH Moseley IF MRI of the intraorbital optic nerve in patients with autosomal dominant optic atrophy. Neuroradiology 2000;42:180–3 10772138 10.1007/s002340050041

[142] Delporto G Vingolo EM Steindl K Forte R Iannaccone A Rispoli E Pannarale MR Clinical heterogeneity of dominant optic atrophy – the contribution of visual function investigations to diagnosis. Graefes Arch Clin Exp Ophthalmol 1994;232:717–27 7890185 10.1007/BF00184274

[143] Holder GE Votruba M Carter AC Bhattacharya SS Fitzke FW Moore AT Electrophysiological findings in dominant optic atrophy (DOA) linking to the OPA1 locus on chromosome 3q 28-qter. Doc Ophthalmol 1998;95:217–28 10532406 10.1023/a:1001844021014

[144] Alexander C Votruba M Pesch UEA Thiselton DL Mayer S Moore A Rodriguez M Kellner U Leo-Kottler B Auburger G Bhattacharya SS Wissinger B OPA1, encoding a dynamin-related GTPase, is mutated in autosomal dominant optic atrophy linked to chromosome 3q28. Nat Genet 2000;26:211–5 11017080 10.1038/79944

[145] Delettre C Lenaers G Griffoin JM Gigarel N Lorenzo C Belenguer P Pelloquin L Grosgeorge J Turc-Carel C Perret E Astarie-Dequeker C Lasquellec L Arnaud B Ducommun B Kaplan J Hamel CP Nuclear gene OPA1, encoding a mitochondrial dynamin-related protein, is mutated in dominant optic atrophy. Nat Genet 2000;26:207–10 11017079 10.1038/79936

[146] Davies V Votruba M Focus on molecules: the OPA1 protein. Exp Eye Res 2006;83:1003–4 16563384 10.1016/j.exer.2005.11.021

[147] Newman NJ Biousse V Hereditary optic neuropathies. Eye 2004;18:1144–60 15534600 10.1038/sj.eye.6701591

[148] Fuhrmann N Alavi MV Wissinger B Genomic rearrangements in the OPA1 gene are frequent in patients with autosomal dominant optic atrophy (poster 5387). *The Association for Research in Vision and Ophthalmology (ARVO) Annual Meeting*. Fort Lauderdale, Florida, 2008

[149] Costeff H Gadoth N Apter N Prialnic M Savir H A familial syndrome of infantile optic atrophy, movement disorder, and spastic paraplegia. Neurology 1989;39:595–7 2494568 10.1212/wnl.39.4.595

[150] Anikster Y Kleta R Shaag A Gahl WA Elpeleg O Type III 3-methylglutaconic aciduria (optic atrophy plus syndrome, or Costeff optic atrophy syndrome): identification of the OPA3 gene and its founder mutation in Iraqi Jews. Am J Hum Genet 2001;69:1218–24 11668429 10.1086/324651 PMC1235533

[151] Kleta R Skovby F Christensen E Rosenberg T Gahl WA Anikster Y 3-methylglutaconic aciduria type III in a non-Iraqi-Jewish kindred: clinical and molecular findings. Mol Genet Metab 2002;76:201–6 12126933 10.1016/s1096-7192(02)00047-1

[152] Reynier P Amati-Bonneau P Verny C Olichon A Simard G Guichet A Bonnemains C Malecaze F Malinge MC Pelletier JB Calvas P Dollfus H Belenguer P Malthiery Y Lenaers G Bonneau D OPA3 gene mutations responsible for autosomal dominant optic atrophy and cataract. J Med Genet 2004;41 e110 10.1136/jmg.2003.016576 PMC1735897 15342707

[153] Verny C Amati-Bonneau P Dubas F Malthiery Y Reynier P Bonneau D An OPA3 gene mutation is responsible for the disease associating optic atrophy and cataract with extrapyramidal signs. Revue Neurologique 2005;161:451–4 15924081 10.1016/s0035-3787(05)85075-1

[154] Davies VJ Powell KA White KE Yip W Hogan V Hollins AJ Davies JR Piechota M Brownstein DG Moat SJ Nichols PP Wride MA Boulton ME Votruba M A missense mutation in the murine Opa3 gene models human Costeff syndrome. Brain 2008;131:368–80 18222992 10.1093/brain/awm333

[155] Leipnitz G Seminotti B Amaral AU de Bortoli G Solano A Schuck PF Wyse ATS Wannmacher CMD Latini A Wajner M Induction of oxidative stress by the metabolites accumulating in 3-methylglutaconic aciduria in cerebral cortex of young rats. Life Sci 2008;82:652–62 18261750 10.1016/j.lfs.2007.12.024

[156] Olichon A Guillou E Delettre C Landes T Arnaune-Pelloquin L Emorine LJ Mils V Daloyau M Hamel C Amati-Bonneau P Bonneau D Reynier P Lenaers G Belenguer P Mitochondrial dynamics and disease, OPA1. Biochim Biophys Acta Mol Cell Res 2006;1763:500–9 10.1016/j.bbamcr.2006.04.003 16737747

[157] Ferre M Amati-Bonneau P Tourmen Y Malthiery Y Reynier P eOPA1: an online database for OPA1 mutations. Hum Mutat 2005;25:423–8 15832306 10.1002/humu.20161

[158] Pesch UEA Leo-Kottler B Mayor S Jurklies B Kellner U Apfelstedt-Sylla E Zrenner E Alexander C Wissinger B OPA1 mutations in patients with autosomal dominant optic atrophy and evidence for semi-dominant inheritance. Hum Mol Genet 2001;10:1359–68 11440988 10.1093/hmg/10.13.1359

[159] Schimpf S Schaich S Wissinger B Activation of cryptic splice sites is a frequent splicing defect mechanism caused by mutations in exon and intron sequences of the OPA1 gene. Hum Genet 2006;118:767–71 16323009 10.1007/s00439-005-0096-7

[160] Schimpf S Fuhrmann N Schaich S Wissinger B Comprehensive cDNA study and quantitative transcript analysis of mutant OPA1 transcripts containing premature termination codons. Hum Mutat 2008;29:106–12 17722006 10.1002/humu.20607

[161] Marchbank NJ Craig JE Leek JP Toohey M Churchill AJ Markham AF Mackey DA Toomes C Inglehearn CF Deletion of the OPA1 gene in a dominant optic atrophy family: evidence that haploinsufficiency is the cause of disease. J Med Genet 2002;39:e47 12161614 10.1136/jmg.39.8.e47 PMC1735190

[162] Amati-Bonneau P Valentino ML Reynier P Gallardo ME Bornstein B Boissiere A Campos Y Rivera H de la Aleja JG Carroccia R Iommarini L Labauge P Figarella-Branger D Marcorelles P Furby A Beauvais K Letournel F Liguori R La Morgia C Montagna P Liguori M Zanna C Rugolo M Cossarizza A Wissinger B Verny C Schwarzenbacher R Martin MA Arenas J Ayuso C Garesse R Lenaers G Bonneau D Carelli V OPA1 mutations induce mitochondrial DNA instability and optic atrophy plus phenotypes. Brain 2008;131:338–51 18158317 10.1093/brain/awm298

[163] Ferraris S Clark S Garelli E Davidzon G Moore SA Kardon RH Bienstock RJ Longley MJ Mancuso M Rios PG Hirano M Copeland WC DiMauro S Progressive external ophthalmoplegia and vision and hearing loss in a patient with mutations in POLG2 and OPA1. Arch Neurol 2008;65:125–31 18195150 10.1001/archneurol.2007.9 PMC2364721

[164] Hudson G Amati-Bonneau P Blakely EL Stewart JD He LP Schaefer AM Griffiths PG Ahlqvist K Suomalainen A Reynier P McFarland R Turnbull DM Chinnery PF Taylor RW Mutation of OPA1 causes dominant optic atrophy with external ophthalmoplegia, ataxia, deafness and multiple mitochondrial DNA deletions: a novel disorder of mtDNA maintenance. Brain 2008;131:329–37 18065439 10.1093/brain/awm272

[165] Aijaz S Erskine L Jeffery G Bhattacharya SS Votruba M Developmental expression profile of the optic atrophy gene product: OPA1 is not localized exclusively in the mammalian retinal ganglion cell layer. Invest Ophthalmol Vis Sci 2004;45:1667–73 15161824 10.1167/iovs.03-1093

[166] Pesch UEA Fries JE Bette S Kalbacher H Wissinger B Alexander C Kohler K OPA1, the disease gene for autosomal dominant optic atrophy, is specifically expressed in ganglion cells and intrinsic neurons of the retina. Invest Ophthalmol Vis Sci 2004;45:4217–25 15505078 10.1167/iovs.03-1261

[167] Ju WK Misaka T Kushnareva Y Nakagomi S Agarwal N Kubo Y Lipton SA Bossy-Wetzel E OPA1 expression in the normal rat retina and optic nerve. J Comp Neurol 2005;488:1–10 15912498 10.1002/cne.20586 PMC1350956

[168] Wang AG Fann MJ Yu HY Yen MY OPA1 expression in the human retina and optic nerve. Exp Eye Res 2006;83:1171–8 16857190 10.1016/j.exer.2006.06.004

[169] Bette S Schlaszus H Wissinger B Meyermann R Mittelbronn M OPA1, associated with autosomal dominant optic atrophy, is widely expressed in the human brain. Acta Neuropathol 2005;109:393–9 15700187 10.1007/s00401-004-0970-8

[170] Kamei S Chen-Kuo-Chang M Cazevieille C Lenaers G Olichon A Belenguer P Roussignol G Renard N Eybalin M Michelin A Delettre C Brabet P Hamel CP Expression of the Opa1 mitochondrial protein in retinal ganglion cells: its downregulation causes aggregation of the mitochondrial network. Invest Ophthalmol Vis Sci 2005;46:4288–94 16249510 10.1167/iovs.03-1407

[171] Guan KL Farh L Marshall TK Deschenes RJ Normal mitochondrial structure and genome maintenance in yeast requires the dynamin-like product of the Mgm1 gene. Curr Genet 1993;24:141–8 7916673 10.1007/BF00324678

[172] Wong ED Wagner JA Scott SV Okreglak V Holewinske TJ Cassidy-Stone A Nunnari J The intramitochondrial dynamin-related GTPase, Mgm1p, is a component of a protein complex that mediates mitochondrial fusion. J Cell Biol 2003;160:303–11 12566426 10.1083/jcb.200209015 PMC2172654

[173] Praefcke GJK McMahon HT The dynamin superfamily: universal membrane tubulation and fission molecules? Nat Rev Mol Cell Biol 2004;5:133–47 15040446 10.1038/nrm1313

[174] Chan DC Mitochondrial fusion and fission in mammals. Annu Rev Cell Dev Biol 2006;22:79–99 16704336 10.1146/annurev.cellbio.22.010305.104638

[175] Olichon A Landes T Arnaune-Pelloquin L Emorine LJ Mils V Guichet A Delettre C Hamel C Amati-Bonneau P Bonneau D Reynier P Lenaers G Belenguer P Effects of OPA1 mutations on mitochondrial morphology and apoptosis: relevance to ADOA pathogenesis. J Cell Physiol 2007;211:423–30 17167772 10.1002/jcp.20950

[176] Zanna C Ghelli A Porcelli AM Karbowski M Youle RJ Schimpf S Wissinger B Pinti M Cossarizza A Vidoni S Valentino ML Rugolo M Carelli V OPA1 mutations associated with dominant optic atrophy impair oxidative phosphorylation and mitochondrial fusion. Brain 2008;131:352–67 18222991 10.1093/brain/awm335

[177] Chen H Chan DC Critical dependence of neurons on mitochondrial dynamics. Curr Opin Cell Biol 2006;18:453–9 16781135 10.1016/j.ceb.2006.06.004

[178] Chan DC Mitochondrial dynamics in disease. N Engl J Med 2007;356:1707–9 17460225 10.1056/NEJMp078040

[179] Chen H McCaffery JM Chan DC Mitochondrial fusion protects against neurodegeneration in the cerebellum. Cell 2007;130:548–62 17693261 10.1016/j.cell.2007.06.026

[180] Kanazawa T The C. elegans Opa1 homologue EAT-3 is essential for resistance to free radicals. PLoS Genet 2008;4:e1000022 18454199 10.1371/journal.pgen.1000022 PMC2265488

[181] Amati-Bonneau P Guichet A Olichon A Chevrollier A Viala F Miot S Ayuso C Odent S Arrouet C Verny C Calmels MN Simard G Belenguer P Wang J Puel JL Hamel C Malthiery Y Bonneau D Lenaers G Reynier P OPA1 R445H mutation in optic atrophy associated with sensorineural deafness. Ann Neurol 2005;58:958–63 16240368 10.1002/ana.20681

[182] Chevrollier A Hereditary optic neuropathies share a common mitochondrial coupling defect. Ann Neurol 2008;63:794–8 18496845 10.1002/ana.21385

[183] Lodi R Tonon C Valentino ML Iotti S Clementi V Malucelli E Barboni P Longanesi L Schimpf S Wissinger B Baruzzi A Barbiroli B Carelli V Deficit of in vivo mitochondrial ATP production in OPA1-related dominant optic atrophy. Ann Neurol 2004;56:719–23 15505825 10.1002/ana.20278

[184] Cipolat S Rudka T Hartmann D Costa V Serneels L Craessaerts K Metzger K Frezza C Annaert W D’Adamio L Derks C Dejaegere T Pellegrini L D’Hooge R Scorrano L De Strooper B Mitochondrial rhomboid PARL regulates cytochrome c release during apoptosis via OPA1-dependent cristae remodeling. Cell 2006;126:163–75 16839884 10.1016/j.cell.2006.06.021

[185] Ishihara N Fujita Y Oka T Mihara K Regulation of mitochondrial morphology through proteolytic cleavage of OPA1. Embo J 2006;25:2966–77 16778770 10.1038/sj.emboj.7601184 PMC1500981

[186] Griparic L Kanazawa T van der Bliek AM Regulation of the mitochondrial dynamin-like protein Opa1 by proteolytic cleavage. J Cell Biol 2007;178:757–64 17709430 10.1083/jcb.200704112 PMC2064541

[187] Frezza C Cipolat S de Brito OM Micaroni M Beznoussenko GV Rudka T Bartoli D Polishuck RS Danial NN De Strooper B Scorrano L OPA1 controls apoptotic cristae remodeling independently from mitochondrial fusion. Cell 2006;126:177–89 16839885 10.1016/j.cell.2006.06.025

[188] Olichon A Baricault L Gas N Guillou E Valette A Belenguer P Lenaers G Loss of OPA1 perturbates the mitochondrial inner membrane structure and integrity, leading to cytochrome c release and apoptosis. J Biol Chem 2003;278:7743–6 12509422 10.1074/jbc.C200677200

[189] Alavi MV Bette S Schimpf S Schuettauf F Schraermeyer U Wehrl HF Ruttiger L Beck SC Tonagel F Pichler BJ Knipper M Peters T Laufs J Wissinger B A splice site mutation in the murine Opa1 gene features pathology of autosomal dominant optic atrophy. Brain 2007;130:1029–42 17314202 10.1093/brain/awm005

[190] Davies VJ Hollins AJ Piechota MJ Yip W Davies JR White KE Nicols PP Boulton ME Votruba M Opa1 deficiency in a mouse model of autosomal dominant optic atrophy impairs mitochondrial morphology, optic nerve structure and visual function. Hum Mol Genet 2007;16:1307–18 17428816 10.1093/hmg/ddm079

[191] Amati-Bonneau P Odent S Derrien C Pasquier L Malthiery Y Reynier P Bonneau D The association of autosomal dominant optic atrophy and moderate deafness may be due to the R445H mutation in the OPA1 gene. Am J Ophthalmol 2003;136:1170–1 14644237 10.1016/s0002-9394(03)00665-2

[192] Li CM Kosmorsky G Zhang K Katz BJ Ge J Traboulsi EI Optic atrophy and sensorineural hearing loss in a family caused by an R445H OPA1 mutation. Am J Med Genet Part A 2005;138A:208–11 10.1002/ajmg.a.30794 16158427

[193] Liguori M A phenotypic variation of dominant optic atrophy and deafness (ADOAD) due to a novel OPA1 mutation. J Neurol 2008;255:127–9 18204809 10.1007/s00415-008-0571-x

[194] Verny C Multiple sclerosis-like disorder in OPA1-related autosomal dominant optic atrophy. Neurology 2008;70:1152–3 18287570 10.1212/01.wnl.0000289194.89359.a1

[195] Stewart JD Hudson G Yu-Wai-Man P Horvath R Maddison P Wright A Blakely EL He LP Griffiths PG Turnbull DM Taylor RW Chinnery PF OPA1 in multiple mitochondrial DNA deletion disorders. Neurology 2008;71:1829–31 19029523 10.1212/01.wnl.0000335931.54095.0a

[196] Toomes C Marchbank NJ Mackey DA Craig JE Newbury-Ecob RA Bennett CP Vize CJ Desai SP Black GCM Patel N Teimory M Markham AF Inglehearn CF Churchill AJ Spectrum, frequency and penetrance of OPA1 mutations in dominant optic atrophy. Hum Mol Genet 2001;10:1369–78 11440989 10.1093/hmg/10.13.1369

[197] Thiselton DL Alexander C Taanman JW Brooks S Rosenberg T Eiberg H Andreasson S Van Regemorter N Munier FL Moore AT Bhattacharya SS Votruba M A comprehensive survey of mutations in the OPA1 gene in patients with autosomal dominant optic atrophy. Invest Ophthalmol Vis Sci 2002;43:1715–24 12036970

[198] Carroll WM Kriss A Baraitser M Barrett G Halliday AM The incidence and nature of visual pathway involvement in Friedreich’s ataxia. A clinical and visual evoked potential study of 22 patients. Brain 1980;103:413–34 7397485 10.1093/brain/103.2.413

[199] Livingstone IR Mastaglia FL Edis R Howe JW Visual involvement in Friedreich’s ataxia and hereditary spastic ataxia. A clinical and visual evoked response study. Arch Neurol 1981;38:75–9 7469840 10.1001/archneur.1981.00510020033003

[200] Chalmers RM Riordan-Eva P Wood NW Autosomal recessive inheritance of hereditary motor and sensory neuropathy with optic atrophy. J Neurol Neurosurg Psychiatry 1997;62:385–7 9120454 10.1136/jnnp.62.4.385 PMC1074097

[201] Zuchner S De Jonghe P Jordanova A Claeys KG Guergueltcheva V Cherninkova S Hamilton SR Van Stavern G Krajewski KM Stajich J Tournev I Verhoeven K Langerhorst CT de Visser M Baas F Bird T Timmerman V Shy M Vance JM Axonal neuropathy with optic atrophy is caused by mutations in mitofusin 2. Ann Neurol 2006;59:276–81 16437557 10.1002/ana.20797

[202] Livingstone IR Mastaglia FL Edis R Howe JW Pattern visual evoked responses in hereditary spastic paraplegia. J Neurol Neurosurg Psychiatry 1981;44:176–8 7217977 10.1136/jnnp.44.2.176 PMC490856

[203] Miyama S Arimoto K Kimiya S Miyama S Complicated hereditary spastic paraplegia with peripheral neuropathy, optic atrophy and mental retardation. Neuropediatrics 2000;31:214–7 11071149 10.1055/s-2000-7462

[204] Makhoul J Cordonnier M Van Nechel C Optic neuropathy in Strumpell-Lorrain disease: presentation of a clinical case and literature review. Bull Soc Belge Ophtalmol 2002;286:9–14 12564312

[205] Bunce C Wormald R Leading causes of certification for blindness and partial sight in England & Wales. Bmc Public Health 2006;6 10.1186/1471-2458-6-58 PMC1420283 16524463

[206] Wiggs JL Genetic etiologies of glaucoma. Arch Ophthalmol 2007;125:30–7 17210849 10.1001/archopht.125.1.30

[207] Buono LM Foroozan R Sergott RC Savino PJ Is normal tension glaucoma actually an unrecognized hereditary optic neuropathy? New evidence from genetic analysis. Curr Opin Ophthalmol 2002;13:362–70 12441838 10.1097/00055735-200212000-00004

[208] Aung T Ocaka L Ebenezer ND Morris AG Krawczak M Thiselton DL Alexander C Votruba M Brice G Child AH Francis PJ Hitchings RA Lehmann OJ Bhattacharya SS A major marker for normal tension glaucoma: association with polymorphisms in the OPA1 gene. Hum Genet 2002;110:52–6 11810296 10.1007/s00439-001-0645-7

[209] Powell B Toomes C Scott S Yeung A Marchbank N Spry P Lumb R Inglehearn C Churchill A Polymorphisms in OPA1 are associated with normal tension glaucoma. Mol Vis 2003;9:460–4 14551537

[210] Mabuchi F Tang S Kashiwagi K Yamagata Z Iijima H Tsukahara S The OPA1 gene polymorphism is associated with normal tension and high tension glaucoma. Am J Ophthalmol 2007;143:125–30 17188046 10.1016/j.ajo.2006.09.028

[211] Abu-Amero KK Morales J Bosley TM Mitochondrial abnormalities in patients with primary open-angle glaucoma. Invest Ophthalmol Vis Sci 2006;47:2533–41 16723467 10.1167/iovs.05-1639

[212] Andrews RM Griffiths PG Johnson MA Turnbull DM Histochemical localisation of mitochondrial enzyme activity in human optic nerve and retina. Br J Ophthalmol 1999;83:231–5 10396204 10.1136/bjo.83.2.231 PMC1722931

[213] Yu Wai Man CY Chinnery PF Griffiths PG Optic neuropathies – importance of spatial distribution of mitochondria as well as function. Med Hypotheses 2005;65:1038–42 16098682 10.1016/j.mehy.2004.10.021

[214] Bristow EA Griffiths PG Andrews RM Johnson MA Turnbull DM The distribution of mitochondrial activity in relation to optic nerve structure. Arch Ophthalmol 2002;120:791–6 12049585 10.1001/archopht.120.6.791

[215] Barron MJ Griffiths P Turnbull DM Bates D Nichols P The distributions of mitochondria and sodium channels reflect the specific energy requirements and conduction properties of the human optic nerve head. Br J Ophthalmol 2004;88:286–90 14736793 10.1136/bjo.2003.027664 PMC1771975

[216] Baloh RH Schmidt RE Pestronk A Milbrandt J Altered axonal mitochondrial transport in the pathogenesis of Charcot-Marie-Tooth disease from mitofusin 2 mutations. J Neurosci 2007;27:422–30 17215403 10.1523/JNEUROSCI.4798-06.2007 PMC6672077

[217] Detmer SA Velde CV Cleveland DW Chan DC Hindlimb gait defects due to motor axon loss and reduced distal muscles in a transgenic mouse model of Charcot-Marie-Tooth type 2A. Hum Mol Genet 2008;17:367–75 17959936 10.1093/hmg/ddm314

[218] Ferreirinha F Quattrini A Pirozzi M Valsecchi V Dina G Broccoli V Auricchio A Piemonte F Tozzi G Gaeta L Casari G Ballabio A Rugarli EI Axonal degeneration in paraplegin-deficient mice is associated with abnormal mitochondria and impairment of axonal transport. J Clin Invest 2004;113:231–42 14722615 10.1172/JCI20138 PMC311437

[219] Rugarli EI Langer T Translating m-AAA protease function in mitochondria to hereditary spastic paraplegia. Trends Mol Med 2006;12:262–9 16647881 10.1016/j.molmed.2006.04.002

[220] Huoponen K Vilkki J Aula P Nikoskelainen EK Savontaus ML A new mtDNA mutation associated with Leber hereditary optic neuroretinopathy. Am J Hum Genet 1991;48:1147–53 1674640 PMC1683111

[221] Howell N Bindoff LA McCullough DA Kubacka I Poulton J Mackey D Taylor L Turnbull DM Leber hereditary optic neuropathy: identification of the same mitochondrial ND1 mutation in six pedigrees. Am J Hum Genet 1991;49:939–50 1928099 PMC1683233

[222] Johns DR Neufeld MJ Park RD An ND-6 mitochondrial DNA mutation associated with Leber hereditary optic neuropathy. Biochem Biophys Res Commun 1992;187:1551–7 1417830 10.1016/0006-291x(92)90479-5

[223] Valentino ML Barboni P Ghelli A Bucchi L Rengo C Achilli A Torroni A Lugaresi A Lodi R Barbiroli B Dotti M Federico A Baruzzi A Carelli V The ND1 gene of complex I is a mutational hot spot for Leber’s hereditary optic neuropathy. Ann Neurol 2004;56:631–41 15505787 10.1002/ana.20236

[224] Kim JY Hwang JM Park SS Mitochondrial DNA C4171A/ND1 is a novel primary causative mutation of Leber’s hereditary optic neuropathy with a good prognosis. Ann Neurol 2002;51:630–4 12112111 10.1002/ana.10177

[225] Leo-Kottler B Luberichs J Besch D Christ-Adler M Fauser S Leber’s hereditary optic neuropathy: clinical and molecular genetic results in a patient with a point mutation at np T11253C (isoleucine to threonine) in the ND4 gene and spontaneous recovery. Graefes Arch Clin Exp Ophthalmol 2002;240:758–64 12271374 10.1007/s00417-002-0494-7

[226] Brown MD Starikovskaya E Derbeneva O Hosseini S Allen JC Mikhailovskaya IE Sukernik RI Wallace DC The role of mtDNA background in disease expression: a new primary LHON mutation associated with Western Eurasian haplogroup J. Hum Genet 2002;110:130–8 11935318 10.1007/s00439-001-0660-8

[227] Mayorov V Biousse V Newman NJ Brown MD The role of the ND5 gene in LHON: characterization of a new, heteroplasmic LHON mutation. Ann Neurol 2005;58:807–11 16240359 10.1002/ana.20669

[228] Howell N Halvorson S Burns J McCullough DA Paulton J When does bilateral optic atrophy become Leber hereditary optic neuropathy? Am J Hum Genet 1993;53:959–63 8213825 PMC1682377

[229] Besch D Leo-Kottler B Zrenner E Wissinger B Leber’s hereditary optic neuropathy: clinical and molecular genetic findings in a patient with a new mutation in the ND6 gene. Graefes Arch Clin Exp Ophthalmol 1999;237:745–52 10447650 10.1007/s004170050307

[230] Zhadanov SI Atamanov VV Zhadanov NI Oleinikov OV Osipova LP Schurr TG A novel mtDNA ND6 gene mutation associated with LHON in a Caucasian family. Biochem Biophys Res Commun 2005;332:1115–21 15922297 10.1016/j.bbrc.2005.05.059

[231] Howell N Bogolin C Jamieson R Marenda DR Mackey DA mtDNA mutations that cause optic neuropathy: how do we know? Am J Hum Genet 1998;62:196–202 9443868 10.1086/301675 PMC1376802

[232] Chinnery PF Brown DT Andrews RM Singh-Kler R Riordan-Eva P Lindley J Applegarth DA Turnbull DM Howell N The mitochondrial ND6 gene is a hot spot for mutations that cause Leber’s hereditary optic neuropathy. Brain 2001;124:209–18 11133798 10.1093/brain/124.1.209

[233] Wissinger B Besch D Baumann B Fauser S Christ-Adler M Jurklies B Zrenner E Leo-Kottler B Mutation analysis of the ND6 gene in patients with Lebers hereditary optic neuropathy. Biochem Biophys Res Commun 1997;234:511–5 9177303 10.1006/bbrc.1997.6660

[234] Fauser S Leo-Kottler B Besch D Luberichs J Confirmation of the 14568 mutation in the mitochondrial ND6 gene as causative in Leber’s hereditary optic neuropathy. Ophthalmic Genet 2002;23:191–7 12324878 10.1076/opge.23.3.191.7881

[235] Parker WD Jr Oley CA Parks JK A defect in mitochondrial electron-transport activity (NADH-coenzyme Q oxidoreductase) in Leber’s hereditary optic neuropathy. N Engl J Med 1989;320:1331–3 2497346 10.1056/NEJM198905183202007

[236] Majander A Huoponen K Savontaus ML Nikoskelainen E Wikstrom M Electron transfer properties of NADH:ubiquinone reductase in the ND1/3460 and the ND4/11778 mutations of the Leber hereditary optic neuroretinopathy (LHON). FEBS Lett 1991;292:289–92 1959619 10.1016/0014-5793(91)80886-8

[237] Larsson NG Andersen O Holme E Oldfors A Wahlstrom J Leber’s hereditary optic neuropathy and complex I deficiency in muscle. Ann Neurol 1991;30:701–8 1763894 10.1002/ana.410300511

[238] Smith PR Cooper JM Govan GG Harding AE Schapira AH Platelet mitochondrial function in Leber’s hereditary optic neuropathy. J Neurol Sci 1994;122:80–3 8195807 10.1016/0022-510x(94)90055-8

[239] Degli Esposti M Carelli V Ghelli A Ratta M Crimi M Sangiorgi S Montagna P Lenaz G Lugaresi E Cortelli P Functional alterations of the mitochondrially encoded ND4 subunit associated with Leber’s hereditary optic neuropathy. FEBS Lett 1994;352:375–9 7926004 10.1016/0014-5793(94)00971-6

[240] Cock HR Cooper JM Schapira AH The 14484 ND6 mtDNA mutation in Leber hereditary optic neuropathy does not affect fibroblast complex I activity [letter]. Am J Hum Genet 1995;57:1501–2 8533781 PMC1801418

[241] Oostra RJ Van Galen MJ Bolhuis PA Bleeker-Wagemakers EM Van den Bogert C The mitochondrial DNA mutation ND6*14,484C associated with Leber hereditary optic neuropathy, leads to deficiency of complex I of the respiratory chain. Biochem Biophys Res Commun 1995;215:1001–5 7488023 10.1006/bbrc.1995.2563

[242] Montagna P Plazzi G Cortelli P Carelli V Lugaresi E Barboni P Fiocchi M Abnormal lactate after effort in healthy carriers of Leber’s hereditary optic neuropathy [letter]. J Neurol Neurosurg Psychiatry 1995;58:640–1 7745422 10.1136/jnnp.58.5.640 PMC1073505

[243] Majander A Finel M Savontaus ML Nikoskelainen E Wikstrom M Catalytic activity of complex I in cell lines that possess replacement mutations in the ND genes in Leber’s hereditary optic neuropathy. Eur J Biochem 1996;239:201–7 8706709 10.1111/j.1432-1033.1996.0201u.x

[244] Hofhaus G Johns DR Hurko O Attardi G Chomyn A Respiration and growth defects in transmitochondrial cell lines carrying the 11778 mutation associated with Leber’s hereditary optic neuropathy. J Biol Chem 1996;271:13155–61 8662757 10.1074/jbc.271.22.13155

[245] Carelli V Ghelli A Ratta M Bacchilega E Sangiorgi S Mancini R Leuzzi V Cortelli P Montagna P Lugaresi E Degli Esposti M Leber’s hereditary optic neuropathy: biochemical effect of 11778/ND4 and 3460/ND1 mutations and correlation with the mitochondrial genotype. Neurology 1997;48:1623–32 9191778 10.1212/wnl.48.6.1623

[246] Cock HR Tabrizi SJ Cooper JM Schapira AH The influence of nuclear background on the biochemical expression of 3460 Leber’s hereditary optic neuropathy. Ann Neurol 1998;44:187–93 9708540 10.1002/ana.410440208

[247] Cock HR Cooper JM Schapira AH Functional consequences of the 3460-bp mitochondrial DNA mutation associated with Leber’s hereditary optic neuropathy. J Neurol Sci 1999;165:10–7 10426140 10.1016/s0022-510x(99)00088-x

[248] Brown MD Trounce IA Jun AS Allen JC Wallace DC Functional analysis of lymphoblast and cybrid mitochondria containing the 3460, 11778, or 14484 Leber’s hereditary optic neuropathy mitochondrial DNA mutation. J Biol Chem 2000;275:39831–6 10976107 10.1074/jbc.M006476200

[249] Baracca A Solaini G Sgarbi G Lenaz G Baruzzi A Schapira AH Martinuzzi A Carelli V Severe impairment of complex I-driven adenosine triphosphate synthesis in Leber hereditary optic neuropathy cybrids. Arch Neurol 2005;62:730–6 15883259 10.1001/archneur.62.5.730

[250] Kerrison JB Arnould VJ Sallum JMF Vagefi MR Barmada MM Li YY Zhu DP Maumenee IH Genetic heterogeneity of dominant optic atrophy, Kjer type - Identification of a second locus on chromosome 18q12.2–12.3. Arch Ophthalmol 1999;117:805–10 10369594 10.1001/archopht.117.6.805

[251] Barbet F Hakiki S Orssaud C Gerber S Perrault I Hanein S Ducroq D Dufier JL Munnich A Kaplan J Rozet JM A third locus for dominant optic atrophy on chromosome 22q. J Med Genet 2005;42:e1 15635063 10.1136/jmg.2004.025502 PMC1735912

[252] Carelli V Schimpf S Valentino ML Fuhrmann N Papke M Schaich S Tippmann S Baumann B Barboni P Ghelli A Bucchi L Lodi R Barbiroli B Liguori R Carroccia R Villanova M Montagna P Baruzzi A Wissinger B Dominant optic atrophy (DOA) and sensorineural hearing loss: clinical, biochemical, spectroscopic and molecular genetic study of a large Italian pedigree linked to a new locus an chromosome 16. Neurology 2007;68:A42

[253] Puccio H Koenig M Friedreich ataxia: a paradigm for mitochondrial diseases. Curr Opin Genet Dev 2002;12:272–7 12076669 10.1016/s0959-437x(02)00298-8

[254] Rouault TA Tong WH Iron-sulphur cluster biogenesis and mitochondrial iron homeostasis. Nat Rev Mol Cell Biol 2005;6:345–51 15803140 10.1038/nrm1620

[255] Zuchner S Vance JM Mechanisms of disease: a molecular genetic update on hereditary axonal neuropathies. Nat Clin Pract Neurol 2006;2:45–53 16932520 10.1038/ncpneuro0071

[256] Casari G Rugarli E Molecular basis of inherited spastic paraplegias. Curr Opin Genet Dev 2001;11:336–42 11377972 10.1016/s0959-437x(00)00199-4

